# Antibiotic Resistance Profile of *Staphylococcus aureus* in Cancer Patients at Laquintinie Hospital in Douala, Littoral Region, Cameroon

**DOI:** 10.1155/2024/5859068

**Published:** 2024-05-15

**Authors:** Michael Francis Kengne, Armelle T. Mbaveng, Victor Kuete

**Affiliations:** Department of Biochemistry, Faculty of Science, University of Dschang, Dschang, Cameroon

## Abstract

Cancer and chemotherapy predispose the patients to various bacterial infections. This study is aimed at isolating and establishing the distribution of antibiotic-resistant *Staphylococcus aureus* from fecal samples in subjects with cancer admitted to the Oncology Department at Laquintinie Hospital in Douala, in the Littoral Region of Cameroon. A cross-sectional study was conducted from October 2021 to March 2023. Cancer and noncancer patients were suffering from *Staphylococcus aureus* infection. The isolation of *Staphylococcus aureus* was based on culture on the specific medium. The Kirby-Bauer disk diffusion method was used for drug susceptibility testing. Of the 507 patients studied, 307 (60.55%) were cancer patients, compared to 200 (39.45%) noncancer patients. *S. aureus* was isolated in 81 (15.97%) participants, among which 62 (76.55%) were cancer patients and 19 (23.45%) were noncancer patients. In the study population, 31.92% of participants had breast cancer, followed by cervical cancer (13.68%) and leukemia (7.17%). *Staphylococcus aureus* isolates showed high resistance rates in cancer patients compared to noncancer patients to amoxicillin-clavulanic acid (AMC, 77.42% versus 31.58%), cefoxitin (FOX, 80.65% versus 63.16%), ciprofloxacin (CIP, 75.81% versus 26.32%), ofloxacin (OFX, 69.35% versus 31.58%), fusidic acid (FUS, 70.97% versus 53.63%), and tetracycline (TET, 85.48% versus 78.95%). *Staphylococcus aureus* showed a significant increase in multidrug-resistant (MDR) and methicillin-resistant (MRSA) phenotypes in cancer patients compared to noncancer patients (*p* < 0.05). The prevalence of MRSA was 76.54%, higher than that of methicillin-sensitive *Staphylococcus aureus* (MSSA) (23.46%). The frequency of MRSA was significantly higher (*p* < 0.001) in cancer patients (80.65%) than in noncancer patients (19.35%). This study showed that there is an association between antibiotic resistance and cancer status. Research and interventions must be focused on the cancer population to combat the appearance of MDR bacteria due to the loss of effectiveness of antibiotics.

## 1. Introduction

There is no doubt that patients in oncology wards are more vulnerable to infections. Cancer and chemotherapy predispose these patients to infections [1]. Infection is commonly encountered in cancer patients, causing disruptions in therapeutic management, prolonged hospitalization, increased healthcare costs, and reduced survival [2]. Cancer patients are immunocompromised and are therefore at high risk of serious opportunistic infections with multidrug-resistant (MDR) bacterial strains [3]. Empirical use of antimicrobials has reduced mortality in patients but has also led to the threat of MDR bacteria [4]. Furthermore, without effective use of antibiotics for the prevention and treatment of infections, the success of major surgery and cancer chemotherapy would be compromised, putting them at even greater risk [5]. *Staphylococcus aureus* is one of the bacteria responsible for infections in susceptible patients, such as those suffering from immune deficiencies. It is an important pathogen, responsible for a variety of diseases in immunocompromised and immunocompetent individuals, due to the large number of toxins and other virulence determinants it produces. It is a Gram-positive bacterium present in the environment (air, soil, water, food, furniture, and materials) and lives in a commensal state on the skin and mucous membranes of human and animal organisms from birth [6]. The reservoir of *S. aureus* is essentially human; it can be isolated, particularly in warm and humid areas of the body such as the nasal cavity, oropharynx, axillary hollows, perineum, and digestive tract [7]. The human intestinal tract harbors many bacteria, including *S. aureus*, which is a potential source of endogenous and exogenous staphylococcal infections [8]. Stool samples can certainly be a significant source of environmental contamination and have been identified as a possible source of antibiotic-resistant *S. aureus*, particularly methicillin-resistant *Staphylococcus aureus* (MRSA) [9]. A report from the Centers for Disease Control and Prevention's (CDCP) National Nosocomial Infection Surveillance System (NNISS) (2013-2015) showed that MRSA in India and the United States accounts for more than 60% of infections in intensive care units. *Staphylococcus aureus* is therefore responsible for nosocomial infections in intensive care units (ICU) [10].

To our knowledge, very few studies have focused on the intestinal reservoir of MRSA. Similarly, there has been no in-depth study regarding the frequency of antibiotic-resistant *S. aureus* from fecal samples from cancer subjects has been reported in medical studies in Cameroon. In this study, we hypothesized that the stools of subjects with cancer could contain strains of MRSA and could serve as a potential source of dissemination of MRSA in the Littoral Region of Cameroon. The aim of this study was therefore to determine the frequency of antibiotic-resistant *Staphylococcus aureus* in fecal samples of subjects suffering from cancer compared to noncancer patients at the Laquintinie Hospital in Douala, in the Littoral Region of Cameroon.

## 2. Materials and Methods

### 2.1. Study Framework

This is a cross-sectional epidemiological study over a period of one and a half years (from October 2021 to March 2023) covering all patients suffering from cancer, regardless of age, who came for consultation in the oncology department. From the Laquintinie Hospital in Douala (Littoral Region of Cameroon), eighty-one (81) patients in whom the presence of *Staphylococcus aureus* was detected in stools were included in this study. Four hundred and twenty-six (426) were excluded. Human immunodeficiency virus- (HIV-) positive patients, patients on antibiotic treatment, and participants with positive serology for hepatitis B and C were not included in this study.

### 2.2. Biological Material

Stool samples were collected from 507 participants who visited the hospital for bacteriological examination and with their informed consent. Sociodemographic data (age, marital status, type of cancer, stage of disease, profession under anticancer treatment or not, type of protocol, and duration of illness) were collected. Duplicates were systematically eliminated.

### 2.3. Sample Collection

In this study, 507 stool samples were collected under aseptic conditions before antibiotic therapy and processed within two hours of receipt. The spatula container was placed in a sealed plastic bag, and patients washed their hands well with soap and water. All the leftover stools were flushed down the toilet. The sample was returned to the laboratory as soon as possible, and microbiological analyses were carried out immediately. These stool samples were used for microbiological analyses.

### 2.4. Isolation and Identification of *Staphylococcus aureus* from Stools

Clinical samples were inoculated onto mannitol salt agar (MSA) plates; they were incubated at 37°C for 24 h. All colonies from the primary culture were purified by subculture on freshly prepared MSA medium and incubated at 37°C for 24 to 48 h [11]. The smear was prepared from the isolated culture on a clean, grease-free, microscopic glass slide and stained with the Gram staining method. The colored smear was observed under a microscope. The smear revealed spherical Gram-positive cells arranged in irregular clusters resembling grapes.

The tube coagulase test was performed by mixing bacterial colonies in a volume of 250 *μ*L of plasma in a small test tube and incubating for 24 h. As bacteria multiply in the plasma, they secrete staphylocoagulase which reacts with plasma globulin factor (coagulase reaction factor) to form staphylothrombin (staphylocoagulase + prothrombin). Staphylothrombin then catalyzes the degradation of fibrinogen into insoluble fibrin which then forms a clot, and we speak of coagulase-positive *Staphylococcus* which refers to *Staphylococcus aureus*. The catalase test was also carried out by placing a drop of hydrogen peroxide (H_2_O_2_) on a slide and then placing it in contact with an isolated colony, collected directly with a single-use plastic loop. If oxygen bubbles form, the bacteria have catalase. These biochemical tests have been performed to confirm *S. aureus* using coagulase-positive and catalase-positive cocci, and they produce yellowish colonies on MSA [12, 13].

### 2.5. Antibiotic-Sensitivity Testing

The susceptibility of isolates to different antimicrobial agents was carried out by the disk diffusion method using commercial disks and interpreted according to EUCAST in 2014 [14]. The results were recorded as sensitive (S), intermediate (I), and resistant (R). Pure colonies of *S. aureus* cultures were inoculated into peptone water and incubated at 37°C to obtain a turbidity equal to 0.5 on the McFarland scale (10^8^ CFU/mL). A sterile swab was dipped into the inoculation, and the excess was removed by squeezing the swab on the sides of the tube. The entire surface of the Mueller-Hinton agar was swabbed. The inoculation was allowed to dry for 15 minutes, and then the antibiotic disks were applied to the media. The Petri dishes containing inoculation and antibiotic disks were incubated at 37°C and examined after 18 to 24 h. Antimicrobial agents tested for *S. aureus* were cefoxitin (FOX, 30 *μ*g), amoxicillin-clavulanic acid (AMC, 10 *μ*g), erythromycin (ERY, 15 *μ*g), amikacin (AMI, 30 *μ*g), gentamicin (GEN, 30 *μ*g), ciprofloxacin (CIP, 5 *μ*g), ofloxacin (OFX, 5 *μ*g), trimethoprim-sulfamethoxazole (COT, 10 *μ*g), tetracycline (TET, 10 *μ*g), nitrofurantoin (NIT, 30 *μ*g), and fusidic acid (FUS, 30 *μ*g). Methicillin sensitivity was tested using a 30 *μ*g cefoxitin disk. An inhibition diameter of <25 mm was considered resistant to methicillin [15]. All raw data on the susceptibility testing as well as the patient's information are available in the Supplementary file (available here).

### 2.6. Ethical Approval

Ethical authorizations were obtained from the National Research Ethics Committee for Human Science (CNERSH), Yaoundé, Cameroon, that delivered an ethical clearance (with reference number: 2022/09/129/CE/CNERSH/SP) and from the institutional ethics committee of the University of Douala (CIE-UD) (with reference number: 3127/CEI-UDo/06/2022/T). We obtained a research certificate from the University of Dschang as well as a research authorization from the Laquintinie Hospital in Douala. All participants were informed of the study objectives, procedures, potential harms and benefits, and costs, as well as the finality of the study. Each patient signed an informed consent form, thereby agreeing to participate in the study. Subsequently, a questionnaire was submitted to them, and sample collection was carried out according to scientific and ethical standards. All results were coded and kept confidential.

### 2.7. Processing and Statistical Analysis of Data

In establishing the percentages of resistance of the different bacterial species, the results were described as “intermediate,” “resistant,” and “susceptible.” The data collected were analyzed by Epi Info™ version 7.2.2.6 (CDC, 1600 Clifton Road, Atlanta). The chi-square (*χ*^2^) test was used to assess the relationship between antimicrobial resistance and specific variables. Crude ORs and exact 95% CIs were calculated to assess the potential relationship between prior antibiotic therapy exposure and multidrug resistance in *Staphylococcus aureus*. Two-tailed Fisher's exact tests were used to compare proportions, and a *p* value < 0.05 was considered statistically significant. Bivariate logistic regression was used to assess whether prior antibiotic exposure was a risk factor for multidrug-resistant *Staphylococcus aureus*.

## 3. Results

### 3.1. Sociodemographic Characteristics of the Study Population

Out of a total of 507 participants, 307 were cancer patients, and 200 were noncancer patients. The mean age of the total study population was 46.38 ± 15.81 years, and that of cancer patients was 49.54 ± 14.65 years. In noncancer patients, the mean age was 41.53 ± 16.33 years. There was a significant difference between the mean age of cancer patients and the mean age of noncancer patients (*p* value < 0.05).  Table 1 shows that 190 (61.89%) cancer patients are in a relationship compared to 119 (59.50%) noncancer patients. Similarly, 84 (27.36%) of cancer patients were bachelors, compared to 69 (34.50%) of noncancer patients. There is a significant difference in the distribution of different age groups. A significant number of cancer patients were observed in the age groups ranging from 41 to 50 years (30.62%), followed by those above 60 years (27.69%) and 51 to 56 years (20.20%), respectively.

### 3.2. Frequency of Different Types of Cancer and Distribution of *Staphylococcus aureus* Isolated according to Different Age Groups

**Figure 1:**
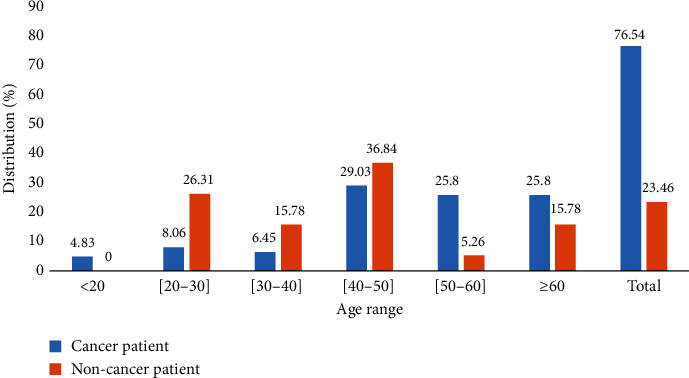
Distribution of *Staphylococcus aureus* isolated according to different age groups.

Table 2 shows the frequency of different types of cancer. In the study of the population, 98 (31.91%) of the participants had breast cancer, followed by cervical cancer with a frequency of 42 (13.68%). Esophagus cancer represented a frequency of 1.63%.  Table 3 shows that 180 patients suffering from cancer had a level of tumor progression at stage 4, i.e., a frequency of 58.63%, followed by stage 3 with 82 patients, i.e., a percentage of 26.71. Stage 2 is less represented with a percentage of 0.65. Furthermore, 188 (61.24%) cancer patients were undergoing anticancer chemotherapy. Out of 507 participants, *S. aureus* was isolated from 81 (15.97%) participants, including 62 (76.54%) from cancer patients and 19 (21.85%) from noncancer patients (Figure 1). In contrast, we isolated more *S. aureus* from cancer and noncancer patients in the age groups of 40 to 49 years (29.03%, 36.84%) and 50 to 59 years (25.58%, 5.26%), respectively.

### 3.3. Different Risk Factors for Resistance in the Study Groups

Table 4 shows the different risk factors for resistance in the study groups. It can be seen that the number of cancer patients who had been exposed to antibiotics in the last six months (*n* = 203) was significantly higher (*p* < 0.001) than the number of noncancer patients (*n* = 56). Of the 307 cancer patients recruited in this study, 180 (58.63%) were hospitalized in the oncology department and 127 (41.37%) were recruited either in consultation in the oncology department or during their chemotherapy treatment. The 200 noncancer patients were mainly recruited from outpatient departments. It should also be noted that 134 or 43.64% of cancer patients had been hospitalized once or twice in the previous year, compared with 30 or 15% of noncancer patients. Many cancer patients, 69 or 22.47%, had been hospitalized three to five times in the past year, compared with only 1 or 0.50% of noncancer patients.

### 3.4. *Staphylococcus aureus* Bacterial Infections and Antibiotic Resistance Profile

The susceptibility of the *S. aureus* isolates obtained from thirteen different antibiotics was evaluated in this study (Table 5). It appears that *S. aureus* presents high resistance rates in cancer patients compared to noncancer patients to AMC (77.42% versus 31.58%), FOX (80.65% against 63.16%), CIP (75.81% against 26.32%), OFX (69.35% against 31.58%), FUS (70.97% against 53.63%), TET (85.48% against 78.95%), and NIT (93.55% against 36.84%). Contrary to the above, the resistance rates of *S. aureus* to AMX (70.97% versus 89.47%), ERY (85.48% versus 89.47%), and COT (54.84% versus 57.89%) were lower among participants suffering from cancer than those not suffering from cancer.

### 3.5. Frequency of Occurrence of Multidrug-Resistant (MDR) and Methicillin-Resistant *Staphylococcus aureus* (MRSA)

**Figure 2:**
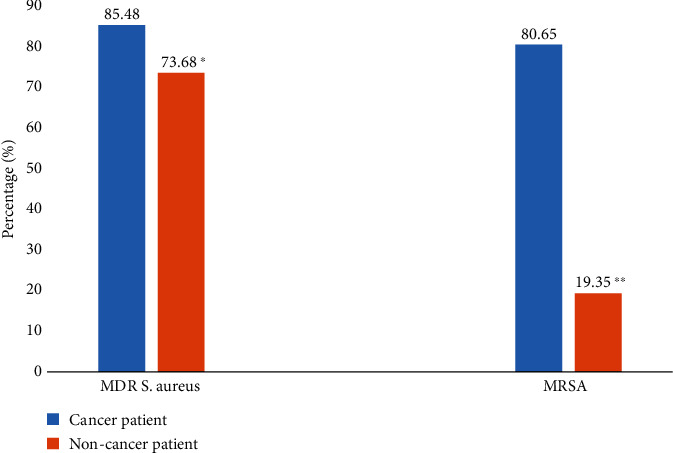
Frequency of occurrence of multidrug-resistant (MDR) and methicillin-resistant *Staphylococcus aureus* (MRSA) bacteria isolated from cancer and noncancer patients. ^∗^*p* = 0.131; ^∗∗^*p* < 0.001.

Figure 2 shows the frequency of the occurrence of multidrug resistance in different isolates in cancer and noncancer patients. *S. aureus* isolates showed high multidrug resistance (85.48%) in cancer patients compared to noncancer patients (73.68%). It is clear that *S. aureus* isolates showed a significant increase in MDR and MRSA phenotypes in cancer patients compared to noncancer patients (*p* < 0.05). Of the 81 *Staphylococcus aureus* isolates, the prevalence of MRSA was 76.54%, higher than that of methicillin-sensitive *Staphylococcus aureus* (MSSA) (23.46%). The frequency of MRSA was significantly higher (*p* < 0.001) in cancer patients (80.65%) than in noncancer patients (19.35%).

### 3.6. Association of Prior Exposure to Antibiotic Therapy in the Last Six Months with Multidrug Resistance of *Staphylococcus aureus*

Table 6 shows the association of prior exposure to antibiotic therapy in the last six months with multidrug resistance of *S. aureus* in cancer and noncancer patients. It was found that cancer patients who had been exposed to a prior course of antibiotic therapy within the last six months were more likely to have an elevated *S. aureus* multidrug resistance profile than cancer patients who had not been previously exposed (OR = 2.44; 95% CI: 0.51-11.63; *p* = 0.233; Table 6). This table also shows that noncancer patients who had prior exposure to antibiotic therapy in the last six months were slightly more likely to have an elevated *S. aureus* multidrug resistance profile than noncancer patients with no prior exposure (OR = 1.60; 95% CI: 0.13-19.09; *p* = 0.602; Table 6). No statistically significant association was found between prior exposure to antibiotic therapy in the past year and the risk of multidrug resistance of *S. aureus* (*p* > 0.05). Nevertheless, cancer patients who had been exposed to a prior course of antibiotic therapy in the last six months had a 2.44-fold increased risk of developing multidrug-resistant *S. aureus* compared with noncancer patients who had just a 1.60-fold increased risk.

### 3.7. Effect of Anticancer Treatment on the Resistance Profile of *Staphylococcus aureus*

**Figure 3:**
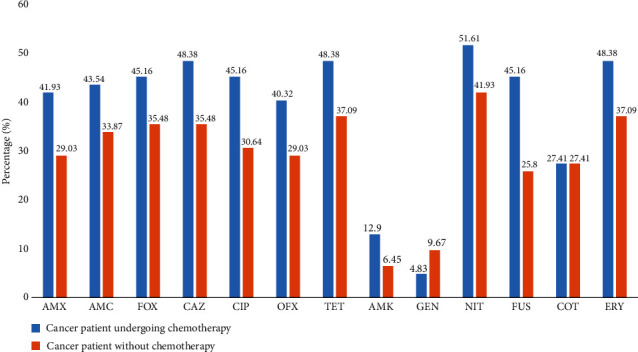
Effect of anticancer treatment on the resistance profile of *Staphylococcus aureus*. AMX: amoxicillin; AMC: amoxicillin-clavulanic acid; CAZ: ceftazidime; FOX: cefoxitin; AMK: amikacin; GEN: gentamicin; TET: tetracycline; CIP: ciprofloxacin; NIT: nitrofurantoin; OFX: ofloxacin; COT: trimethoprim-sulfamethoxazole; FUS: fusidic acid; ERY: erythromycin.

Figure 3 shows the frequency of the occurrence of drug resistance in different isolates in cancer patients depending on anticancer treatment. It appears that the resistance rates were significantly high (with *p* < 0.05) in patients suffering from cancer who underwent courses of chemotherapy compared to patients suffering from cancer without anticancer treatment to AMX (41.93% against 29.03%), CIP (45.16% against 30.64%), OFX (40.32% against 29.03%), and NIT (51.61% against 41.93%).

## 4. Discussion

The bacteria frequently responsible for nosocomial infections in Cameroon, particularly in the city of Douala, are Gram-positive cocci including *S. aureus* [16, 17]. Staphylococci are bacteria involved in numerous pathologies of varying severity, and *Staphylococcus aureus* ranks first among the Gram-positive opportunistic cocci responsible for nosocomial infections [18–20]. It is now a human health problem that requires multidisciplinary efforts at a global level due to the emergence of MRSA pandemics in hospitals [21]. *S. aureus* is a particularly important pathogen in immunocompromised patients, particularly those with neutropenia. *S. aureus* has emerged as an ever-increasing problem due to its increasing antibiotic resistance. This study determined the susceptibility profiles of *S. aureus* strains isolated from the stools of cancer and noncancer patients at Laquintinie Hospital in Douala to provide physicians with up-to-date information on local antimicrobial resistance data for this pathogenic agent.

Out of 507 participants, *S. aureus* was isolated in 81 (15.97%) participants, i.e., 62 (76.54%) in cancer patients and 19 (21.85%) in noncancer patients. A high number of *S. aureus* were isolated from cancer and noncancer patients in the age groups of 41 to 50 years (29.03%, 36.84%) and 51 to 60 years (25.58%, 5.26%), respectively. However, the frequency of 76.55% in cancer patients and 23.45% in noncancer patients observed in our study was much higher than the 51.5.6% obtained in Ethiopia by Worku et al. [22] and the 12.6% obtained in India [23].

Antimicrobial susceptibility testing revealed that *S. aureus* isolates had higher resistance in cancer patients compared to noncancer patients to AMC (77.42% vs. 31.58%), FOX (80.65% against 63.16%), CIP (75.81% against 26.32%; *p* < 0.001; chi‐square = 15.50), TET (85.48% against 78.95%), and NIT (93.55% against 36.84%). Contrary to the above, the resistance rates of *S. aureus* to AMX (70.97% versus 89.47%), ERY (85.48% versus 89.47%), and COT (54.84% versus 57.89%) were lower among participants with cancer than those without cancer, while AMK had the highest overall sensitivity rate of 78.95%, followed by GEN (89.47%). The sensitivity profiles of AMK and GEN were different in patients with cancer and in patients without cancer. These results show a marked increase in the resistance of *S. aureus* isolates in cancer patients compared to noncancer patients. This result is comparable to the results of Njoungang et al. in 2015 at the Military Hospital of Yaoundé; however, this good sensitivity towards antibiotics from the aminoglycoside family (GEN and AMK) could be explained by the limited use and unavailability on the local market of these molecules [24]. Low antibiotic activity was observed for FOX and AMC in the different study groups. This reduction in sensitivity could be the consequence of the hyperproduction of penicillinase responsible for the hydrolysis of penicillin M and cephalosporins; the most probable cause of all these mechanisms would be the genetic plasticity of *S. aureus* and certainly the empirical or inappropriate consumption of these antibiotics [25]. This study also showed a high rate of MDR and MRSA, and therefore it is necessary to improve surveillance systems to reduce the spread of these multidrug-resistant bacteria [21]. The frequencies vary from one country to another but are generally high: 16% in Senegal and Niger, 20% to 47% in Nigeria, 36% in Benin, and 35.7% in Togo [26]. The high rate of MDRs shown in this study, particularly in cancer patients, can be explained by the fact that almost two-thirds (66.12%) of these patients had been previously exposed to antibiotics in the last six months. The use of antibiotics here makes it possible to prevent infectious events and treat bacterial infections, which are very common in cancer patients [3]. This high rate of MDRs is also often correlated with numerous episodes of hospitalization and prolonged hospital stays in the oncology unit of cancer patients, which ultimately reduces the patient's survival time.

This high rate of multidrug resistance as well as the high rate of resistance observed in cancer patients undergoing anticancer treatment can be explained by the fact that, regarding their action on DNA replication, anticancer chemotherapy could increase the basal mutation rate in bacteria and increase the risk of selection of antibiotic-resistant mutants, as cancer chemotherapy can increase mutation rates of bacteria, accelerating the spread of bacterial resistance [27].

Although this study presents data on cancer in patients for whom information on *S. aureus* resistance is extremely limited, we must recognize certain limitations. The generalizability of the data could be compromised by sampling bias. The lack of adjustment for details such as prior use of monotherapy, dual or triple antibacterial therapy, considering the number of days spent on antibiotic therapy, the risk factor prior exposure to antibiotic therapy in the past year, and recollection of MDR is a limitation of this study. The lack of adjustment between the total number of days spent in the hospital in the past year by patients and recollection of MDR is another limitation of the study. In addition, the data should not be generalized to the whole country. Despite the limitations highlighted, we remain convinced that this study presents vital information on the intestinal carriage of multidrug-resistant MRSA in cancer patients. Therefore, the data have important implications for the quality of patient care and infection control practices. In addition, the cross-sectional design of this study limits the ability to examine causal relationships between variables.

## 5. Conclusions

The present study revealed that the distribution of *S. aureus* infections as well as resistant phenotypes were higher in cancer patients. The need for appropriate use of antimicrobials to stop, or at least limit, the spread of resistance is suggested in the care of cancer patients and the general population. We suggest that clinicians should focus more on intestinal colonisation by multidrug-resistant methicillin-resistant *Staphylococcus aureus* (MRSA) in cancer patients and take into account the epidemiological characteristics of local resistance patterns when initiating antimicrobial therapy.

## Figures and Tables

**Table 1:** Frequency of sociodemographic characteristics of cancer patients and healthy patients at Laquintinie Hospital in Douala.

Variable	Cancer patients (*n* = 307) (%)	Noncancer patients (*n* = 200) (%)	*p* value
Sex			
Male	109 (35.5)	83 (41.5)	0.102
Female	198 (64.50)	117 (58.5)	
Age group per year			
<20 years	11 (3.58)	12 (6.00)	<0.001
21-30 years	16 (5.21)	36 (18.00)	
31-40 years	39 (12.70)	49 (24.50)	
41-50 years	94 (30.62)	46 (23.00)	
51-60 years	62 (20.20)	27 (13.50)	
>60 years old	85 (27.69)	30 (15.00)	
Marital status			
Bachelor	84 (27.36)	69 (34.50)	0.117
Bride	190 (61.89)	119 (59.50)	
Widower	31 (10,10)	12 (6.00)	
Divorced	00 (0.00)	02 (0.65)	
Level of study			
Analphabet	10 (5.00)	48 (15.64)	<0.001
Primary school	31 (15.5)	53 (17.26)	
Secondary school	120 (60.00)	176 (57.33)	
Higher education	39 (19.50)	30 (9.77)	
Occupation			
Student	17 (5.54)	35 (17.50)	<0.001
Household	115 (37.46)	45 (22.50)	
Jobless	33 (10.75)	9 (4.50)	
Civil servant	52 (16.94)	38 (19.00)	
Private sector	90 (29.32)	73 (36.50)	

**Table 2:** Frequency of different types of cancer.

Type of cancer	Number	Percentage
Breast	98	31.92%
Cervical	42	13.68%
Leukemia	22	7.17%
Colorectal	19	6.19%
Osteosarcoma	18	5.86%
Stomach	15	4.89%
Liver	14	4.56%
Pancreas	14	4.56%
Prostate	14	4.56%
Kaposi sarcoma	14	4.56%
Cavum	10	3.26%
Lung	8	2.61%
Cholangiocarcinoma	8	2.61%
Ovary	6	1.95%
Esophagus	5	1.63%
Total	307	100.00%

**Table 3:** Different characteristics of the disease.

Variable	Characteristics of the disease	Cancer patients (*n* = 307) (%)
Stage of disease	Stage 2	2 (0.65)
	Stage 3	82 (26.71)
	Stage 4	180 (58.63)
	Unknown stage	43 (14.01)

Chemotherapy	Yes	188 (61.24)
	No	119 (38.76)

Type of chemotherapy	Adjuvant	38 (20.21)
	Neoadjuvant	57 (30.32)
	Exclusive	73 (38.83)
	Palliative	20 (10.64)

Duration of illness	6 months	86 (28.10)
	12 months	95 (31.05)
	>12 months	125 (40.85)

**Table 4:** Different risk factors for resistance in the study groups.

	Cancer patients	Noncancer patients	*X* ^2^ (*p* value)
Exposure to antibiotics in the past six months			
Yes	203 (66.12%)	56 (28%)	70.34 (<0.001)
No	104 (33.88%)	144 (72%)	
Hospitalized patients			
Yes	180 (58.63%)	0 (0.00%)	181.81 (<0.001)
No	127 (41.37%)	200 (100%)	
Number of hospitalizations during the past year			
1–2 times	134 (43.64%)	30 (15%)	14.08 (<0.001)
3–5 times	69 (22.47%)	1 (0.50%)	9.87 (<0.001)
>5 times	10 (3.25%)	0 (0.00%)	1.51 (0.124)
Number of days last hospitalized			
3–5 days	79 (25.73%)	ND	N/A
6–8 days	65 (21.17%)	ND	
>8 days	33 (10.74%)	ND	

ND: not defined; N/A: not applicable; *X*^2^: chi-square.

**Table 5:** Antibiotic resistance profile of *Staphylococcus aureus* isolates from cancer and noncancer patients.

Antibiotics		Patients, *Staphylococcus aureus* profile, and statistical analyses			
		Cancer + (*n* = 62) (%)	Cancer - (*n* = 19) (%)	*X* ^2^	*p* value
AMX	S	18 (29.03)	2 (10.53)	1.17	0.05
	I	0 (0.00)	0 (0.00)		
	R	44 (70.97)	17 (89.47)		

AMC	S	14 (22.58)	13 (68.42)	11.76	<0.0 01
	I	0 (0.00)	0 (0.00)		
	R	48 (77.42)	6 (31.58)		

FOX	S	12 (19.35)	7 (36.84)	1.14	0.06
	I	0 (0.00)	0 (0.00)		
	R	50 (80.65)	12 (63.16)		

CAZ	S	10 (16.13)	5 (26.32)	1.01	0.170
	R	52 (83.87)	14 (73.68)		

AMK	S	50 (80.65)	15 (78.95)	0.85	0.551
	R	12 (19.35)	4 (21.05)		

GEN	S	53 (85.48)	17 (89.47)	0.78	0.353
	R	9 (14.52)	2 (10.53)		

CIP	S	13 (20.97)	12 (63.16)	15.50	<0.0 01
	I	2 (3.23)	2 (10.53)		
	R	47 (75.81)	5 (26.32)		

OFX	S	17 (27.42)	13 (68.42)	10.64	<0.0 01
	I	2 (3.23)	0 (0.00)		
	R	45 (69.35)	6 (31.58)		

FUS	S	17 (27.42)	9 (47.37)	0.79	0.241
	I	1 (1.61)	0 (0.00)		
	R	44 (70.97)	10 (53.63)		

TET	S	9 (14.52)	4 (21.05)	0.345	0.254
	I	0 (0.00)	0 (0.00)		
	R	53 (85.48)	15 (78.95)		

NIT	S	4 (6.45)	11 (57.89)	29.87	<0.0 01
	I	0 (0.00)	1 (5.26)		
	R	58 (93.55)	7 (36.84)		

COT	S	28 (45.16)	8 (42.11)	0.25	0.412
	R	34 (54.84)	11 (57.89)		

ERY	S	9 (14.52)	2 (10.52)	1.02	0.287
	R	53 (85.48)	17 (89.47)		

AMX: amoxicillin; AMC: amoxicillin-clavulanic acid; CAZ: ceftazidime; FOX: cefoxitin; AMK: amikacin; GEN: gentamicin; TET: tetracycline; CIP: ciprofloxacin; NIT: nitrofurantoin; OFX: ofloxacin; COT: trimethoprim-sulfamethoxazole; FUS: fusidic acid; ERY: erythromycin; cancer +: cancer patients; cancer -: noncancer patients; S: sensitive; I: intermediate; R: resistant.

**Table 6:** Association of prior exposure to antibiotic therapy in the last six months with multidrug resistance of *Staphylococcus aureus* following bivariate logistic regression analysis.

	MDR *S. aureus* (*n* = 67) (%)	Non-MDR *S. aureus* (*n* = 14) (%)	OR (95% CI)	*p* value
Cancer patients				
Previous exposure to antibiotic therapy	44 (65.67)	6 (42.85)	2.44 (0.51-11.63)	0.233
No previous exposure to antibiotic therapy	9 (13.43)	3 (21.42)		
Noncancer patients				
Previous exposure to antibiotic therapy	4 (5.97)	1 (7.14)	1.60 (0.13-19.09)	0.602
No previous exposure to antibiotic therapy	10 (14.92)	4 (28.57)		

OR: odds ratio; CI: confidence interval; MDR: multidrug resistance; *S. aureus*: *Staphylococcus aureus*.

## Data Availability

All data generated or analyzed during this study are included in this published article and the supporting file.

## Supplementary Materials

Supplementary Materials Supplementary file (.xls): raw data of the study. S1: participants and their features. S2: patients and cancer treatments. S3: patients, *S. aureus* infection, and antibiogram.
