# Supplementary material for: Antibiotic Resistance Profile of Staphylococcus aureus in Cancer Patients at Laquintinie Hospital in Douala, Littoral Region, Cameroon
Source: Biomed Res Int. 2024 May 15;2024:5859068. doi: 10.1155/2024/5859068 (PMC11111294; doi:10.1155/2024/5859068)
Supplement: Supplementary Materials — Supplementary file (.xls): raw data of the study. S1: participants and their features. S2: patients and cancer treatments. S3: patients, S. aureus infection, and antibiogram. [file 5859068.f1.pdf]

Antibiotic Resistance Profile of *Staphylococcus aureus* in Cancer Patients at Laquintinie Hospital in Douala, Littoral Region, Cameroon

Michael Francis Kengne[a], Armelle T. Mbaveng[a], Victor Kuete[a]***

[a]Department of Biochemistry, Faculty of Science, University of Dschang, Dschang, Cameroon

Corresponding author:

*Email: armbatsa@yahoo.fr; ORCID: https://orcid.org/0000-0003-4178-4967 (Armelle T. Mbaveng)

**Email: kuetevictor@yahoo.fr; ORCID: http://orcid.org/0000-0002-1070-1236 (Victor Kuete)

Other authors emails:

Email: fmkengne@yahoo.com (Michael Francis Kengne)

S1. Participants and their Features

Patients	Patient features					
**Patient Code**	**Sex**	**Age**	**Age range**	**Education level**	**Profession**	**Marital status**
K001	M	30	[30-40[	Secondary school	Private sector	Bachelor
K002	M	54	[50-60[	Primary school	Private sector	Bride
K003	F	50	[50-60[	Secondary school	Household	Bride
K004	F	49	[40-50[	Primary school	Household	Bride
K005	M	31	[30-40[	Higher education	Private sector	Bachelor
K006	M	59	[50-60[	Secondary school	Private sector	Bride
K007	F	67	≥60	Primary school	Household	Widow(er)
K008	M	38	[30-40[	Secondary school	Jobless	Bachelor
K009	F	42	[40-50[	Secondary school	Civil servant	Widow(er)
K010	F	43	[40-50[	Secondary school	Civil servant	Bride
K011	F	41	[40-50[	Secondary school	Household	Bride
K012	M	82	≥60	Analphabet	Private sector	Bride
K013	F	54	[50-60[	Primary school	Household	Widow(er)
K014	M	18	<20	Secondary school	Student	Bachelor
K015	F	33	[30-40[	Higher education	Civil servant	Bride
K016	M	42	[40-50[	Higher education	Civil servant	Bride
K017	M	68	≥60	Primary school	Private sector	Bride
K018	M	66	≥60	Analphabet	Jobless	Bride
K019	M	70	≥60	Analphabet	Private sector	Bride
K020	F	43	[40-50[	Secondary school	Household	Bride
K021	M	61	≥60	Secondary school	Jobless	Bride
K022	M	64	≥60	Higher education	Jobless	Bride
K023	M	57	[50-60[	Secondary school	Jobless	Bride

K024	M	53	[50-60[	Secondary school	Private sector	Bachelor
K025	F	64	≥60	Primary school	Household	Widow(er)
K026	M	29	[20-30[	Secondary school	Private sector	Bachelor
K027	M	75	≥60	Primary school	Jobless	Bride
K028	F	62	≥60	Primary school	Household	Bride
K029	F	58	[50-60[	Secondary school	Household	Bride
K030	F	40	[40-50[	Secondary school	Household	Bachelor
K031	M	65	≥60	Secondary school	Jobless	Bride
K032	F	50	[50-60[	Secondary school	Household	Bride
K033	F	27	[20-30[	Secondary school	Civil servant	Bachelor
K034	F	38	[30-40[	Higher education	Civil servant	Bachelor
K035	F	43	[40-50[	Secondary school	Household	Bride
K036	F	52	[50-60[	Higher education	Household	Bride
K037	F	40	[40-50[	Secondary school	Household	Bride
K038	M	43	[40-50[	Primary school	Jobless	Bride
K039	M	74	≥60	Analphabet	Jobless	Bride
K040	M	75	≥60	Primary school	Jobless	Bride
K041	M	51	[50-60[	Secondary school	Private sector	Widow(er)
K042	F	74	≥60	Analphabet	Household	Widow(er)
K043	F	72	≥60	Analphabet	Household	Widow(er)
K044	F	43	[40-50[	Secondary school	Private sector	Bride
K045	M	56	[50-60[	Secondary school	Private sector	Bride
K046	F	67	≥60	Analphabet	Private sector	Widow(er)
K047	M	43	[40-50[	Secondary school	Private sector	Bride
K048	F	55	[50-60[	Secondary school	Household	Widow(er)
K049	F	50	[50-60[	Secondary school	Household	Bride
K050	M	50	[50-60[	Higher education	Civil servant	Bride
K051	F	42	[40-50[	Secondary school	Private sector	Bachelor
K052	F	45	[40-50[	Secondary school	Household	Bride

K053	F	47	[40-50[	Secondary school	Private sector	Bride
K054	F	63	≥60	Secondary school	Household	Widow(er)
K055	F	10	<20	Primary school	Student	Bachelor
K056	F	69	≥60	Primary school	Private sector	Bride
K057	M	66	≥60	Secondary school	Civil servant	Bride
K058	F	59	[50-60[	Analphabet	Private sector	Widow(er)
K059	M	60	≥60	Secondary school	Civil servant	Bride
K060	F	56	[50-60[	Secondary school	Household	Bride
K061	F	40	[40-50[	Secondary school	Household	Bride
K062	F	38	[30-40[	Primary school	Household	Bride
K063	M	78	≥60	Secondary school	Jobless	Bride
K064	F	60	≥60	Primary school	Private sector	Bride
K065	M	68	≥60	Secondary school	Civil servant	Bride
K066	M	77	≥60	Analphabet	Private sector	Bride
K067	M	39	[30-40[	Higher education	Civil servant	Bride
K068	F	36	[30-40[	Secondary school	Private sector	Bachelor
K069	M	34	[30-40[	Secondary school	Jobless	Bachelor
K070	M	29	[20-30[	Secondary school	Private sector	Bachelor
K071	M	55	[50-60[	Secondary school	Civil servant	Divorced
K072	F	57	[50-60[	Secondary school	Private sector	Divorced
K073	F	41	[40-50[	Secondary school	Household	Bride
K074	F	23	[20-30[	Secondary school	Student	Bachelor
K075	F	65	≥60	Secondary school	Household	Widow(er)
K076	F	75	≥60	Primary school	Household	Widow(er)
K077	M	47	[40-50[	Primary school	Private sector	Bride
K078	M	57	[50-60[	Primary school	Private sector	Bride
K079	F	50	[50-60[	Higher education	Civil servant	Bride
K080	F	41	[40-50[	Secondary school	Jobless	Bachelor
K081	F	60	≥60	Secondary school	Household	Bride

K082	F	51	[50-60[	Secondary school	Household	Bride
K083	F	49	[40-50[	Secondary school	Household	Bride
K084	M	70	≥60	Higher education	Civil servant	Bride
K085	F	44	[40-50[	Secondary school	Household	Bride
K086	M	70	≥60	Secondary school	Jobless	Bride
K087	F	67	≥60	Secondary school	Household	Bride
K088	F	44	[40-50[	Secondary school	Household	Bride
K089	M	75	≥60	Secondary school	Jobless	Bride
K090	M	50	[50-60[	Secondary school	Civil servant	Bride
K091	M	58	[50-60[	Analphabet	Household	Bride
K092	F	37	[30-40[	Secondary school	Household	Bride
K093	F	48	[40-50[	Secondary school	Private sector	Bride
K094	M	33	[30-40[	Secondary school	Private sector	Bachelor
K095	F	50	[50-60[	Secondary school	Private sector	Bride
K096	M	69	≥60	Secondary school	Jobless	Bride
K097	F	48	[40-50[	Secondary school	Household	Bride
K098	F	15	<20	Secondary school	Student	Bachelor
K099	M	58	[50-60[	Secondary school	Jobless	Bride
K100	F	52	[50-60[	Secondary school	Household	Bride
K101	F	29	[20-30[	Secondary school	Civil servant	Bachelor
K102	M	13	<20	Primary school	Student	Bachelor
K103	M	32	[30-40[	Secondary school	Private sector	Bachelor
K104	F	54	[50-60[	Secondary school	Household	Bachelor
K105	F	56	[50-60[	Secondary school	Civil servant	Widow(er)
K106	F	41	[40-50[	Secondary school	Household	Bride
K107	M	11	<20	Primary school	Student	Bachelor
K108	F	44	[40-50[	Secondary school	Civil servant	Bride
K109	F	43	[40-50[	Secondary school	Household	Bride
K110	F	38	[30-40[	Secondary school	Household	Bride

K111	F	12	<20	Primary school	Student	Bachelor
K112	F	41	[40-50[	Secondary school	Household	Bride
K113	M	57	[50-60[	Secondary school	Jobless	Bride
K114	F	38	[30-40[	Secondary school	Civil servant	Bride
K115	F	54	[50-60[	Secondary school	Household	Bride
K116	F	44	[40-50[	Secondary school	Private sector	Bride
K117	F	50	[50-60[	Secondary school	Household	Bride
K118	M	71	≥60	Secondary school	Jobless	Bride
K119	F	68	≥60	Analphabet	Household	Bride
K120	F	61	≥60	Analphabet	Household	Widow(er)
K121	F	60	≥60	Analphabet	Household	Widow(er)
K122	F	49	[40-50[	Secondary school	Civil servant	Bride
K123	F	39	[30-40[	Secondary school	Household	Bride
K124	M	37	[30-40[	Secondary school	Private sector	Bride
K125	F	64	≥60	Secondary school	Household	Bride
K126	F	36	[30-40[	Secondary school	Household	Bachelor
K127	F	45	[40-50[	Secondary school	Civil servant	Bride
K128	F	50	[50-60[	Secondary school	Household	Bride
K129	F	30	[30-40[	Secondary school	Household	Bride
K130	F	45	[40-50[	Secondary school	Private sector	Bachelor
K131	F	47	[40-50[	Primary school	Household	Bride
K132	F	76	≥60	Analphabet	Household	Widow(er)
K133	F	40	[40-50[	Higher education	Civil servant	Bride
K134	F	27	[20-30[	Higher education	Civil servant	Bride
K135	M	80	≥60	Analphabet	Jobless	Bride
K136	F	55	[50-60[	Analphabet	Household	Bride
K137	M	11	<20	Primary school	Student	Bachelor
K138	F	50	[50-60[	Analphabet	Household	Bride
K139	M	10	<20	Primary school	Student	Bachelor

K140	M	63	≥60	Primary school	Private sector	Bride
K141	F	19	<20	Secondary school	Student	Bachelor
K142	M	42	[40-50[	Secondary school	Private sector	Bride
K143	M	32	[30-40[	Analphabet	Jobless	Bride
K144	M	46	[40-50[	Secondary school	Private sector	Bride
K145	F	54	[50-60[	Primary school	Private sector	Bride
K146	F	43	[40-50[	Secondary school	Household	Bachelor
K147	M	58	[50-60[	Primary school	Private sector	Bride
K148	F	72	≥60	Analphabet	Household	Widow(er)
K149	F	49	[40-50[	Secondary school	Household	Bride
K150	F	79	≥60	Analphabet	Household	Widow(er)
K151	F	62	≥60	Primary school	Private sector	Bride
K152	F	66	≥60	Analphabet	Household	Widow(er)
K153	F	66	≥60	Analphabet	Household	Bride
K154	F	51	[50-60[	Primary school	Household	Bachelor
K155	F	46	[40-50[	Secondary school	Civil servant	Bachelor
K156	F	60	≥60	Primary school	Household	Bride
K157	F	68	≥60	Secondary school	Household	Bride
K158	M	61	≥60	Secondary school	Civil servant	Bride
K159	M	45	[40-50[	Secondary school	Jobless	Bachelor
K160	F	47	[40-50[	Secondary school	Household	Bachelor
K161	F	32	[30-40[	Higher education	Civil servant	Bachelor
K162	F	54	[50-60[	Analphabet	Private sector	Bride
K163	F	64	≥60	Analphabet	Household	Bachelor
K164	F	48	[40-50[	Secondary school	Household	Bride
K165	M	43	[40-50[	Secondary school	Private sector	Bride
K166	M	64	≥60	Secondary school	Jobless	Bride
K167	F	60	≥60	Primary school	Household	Bride
K168	M	56	[50-60[	Primary school	Private sector	Bride

K169	M	47	[40-50[	Secondary school	Private sector	Bride
K170	F	56	[50-60[	Secondary school	Household	Bride
K171	F	39	[30-40[	Higher education	Civil servant	Bride
K172	F	31	[30-40[	Secondary school	Household	Bachelor
K173	M	57	[50-60[	Secondary school	Civil servant	Bride
K174	M	54	[50-60[	Secondary school	Jobless	Bride
K175	M	28	[20-30[	Secondary school	Student	Bachelor
K176	M	45	[40-50[	Primary school	Private sector	Bride
K177	M	62	≥60	Primary school	Private sector	Bride
K178	M	62	≥60	Secondary school	Private sector	Bride
K179	M	55	[50-60[	Secondary school	Civil servant	Bride
K180	F	46	[40-50[	Secondary school	Private sector	Bachelor
K181	M	42	[40-50[	Primary school	Private sector	Bride
K182	M	72	≥60	Primary school	Jobless	Bride
K183	F	45	[40-50[	Secondary school	Private sector	Bride
K184	M	69	≥60	Secondary school	Jobless	Bride
K185	F	36	[30-40[	Higher education	Civil servant	Bride
K186	M	41	[40-50[	Secondary school	Household	Bride
K187	F	47	[40-50[	Secondary school	Household	Bride
K188	M	67	≥60	Analphabet	Private sector	Bride
K189	F	63	≥60	Analphabet	Household	Bride
K190	F	66	≥60	Secondary school	Household	Bachelor
K191	M	28	[20-30[	Secondary school	Private sector	Bachelor
K192	M	54	[50-60[	Secondary school	Private sector	Bride
K193	M	41	[40-50[	Secondary school	Private sector	Bride
K194	F	36	[30-40[	Primary school	Civil servant	Bachelor
K195	M	48	[40-50[	Higher education	Civil servant	Bride
K196	F	64	≥60	Analphabet	Household	Bachelor
K197	M	60	≥60	Secondary school	Civil servant	Bride

K198	M	45	[40-50[	Secondary school	Civil servant	Bachelor
K199	M	41	[40-50[	Primary school	Private sector	Bachelor
K200	F	54	[50-60[	Secondary school	Household	Bride
K201	M	37	[30-40[	Secondary school	Private sector	Bachelor
K202	M	39	[30-40[	Secondary school	Private sector	Bachelor
K203	M	43	[40-50[	Primary school	Jobless	Bachelor
K204	F	60	≥60	Primary school	Household	Bachelor
K205	F	43	[40-50[	Secondary school	Civil servant	Bride
K206	F	40	[40-50[	Primary school	Household	Bride
K207	M	44	[40-50[	Analphabet	Private sector	Bride
K208	F	61	≥60	Secondary school	Household	Bride
K209	F	56	[50-60[	Primary school	Household	Bride
K210	F	31	[30-40[	Secondary school	Household	Bachelor
K211	F	62	≥60	Secondary school	Household	Bride
K212	M	52	[50-60[	Higher education	Civil servant	Bride
K213	F	63	≥60	Secondary school	Household	Bride
K214	F	39	[30-40[	Secondary school	Private sector	Bride
K215	F	45	[40-50[	Higher education	Household	Bride
K216	M	44	[40-50[	Higher education	Civil servant	Bride
K217	F	31	[30-40[	Higher education	Civil servant	Bride
K218	F	47	[40-50[	Higher education	Civil servant	Bride
K219	F	36	[30-40[	Secondary school	Private sector	Bachelor
K220	F	28	[20-30[	Secondary school	Private sector	Bachelor
K221	F	47	[40-50[	Secondary school	Household	Bride
K222	F	42	[40-50[	Secondary school	Private sector	Bride
K223	F	72	≥60	Primary school	Household	Widow(er)
K224	F	49	[40-50[	Secondary school	Household	Bride
K225	F	36	[30-40[	Secondary school	Private sector	Bride
K226	F	45	[40-50[	Secondary school	Household	Bride

K227	F	75	≥60	Analphabet	Household	Widow(er)
K228	F	29	[20-30[	Secondary school	Household	Bride
K229	M	46	[40-50[	Primary school	Private sector	Bride
K230	F	45	[40-50[	Secondary school	Private sector	Bride
K231	F	54	[50-60[	Secondary school	Private sector	Bride
K232	F	47	[40-50[	Secondary school	Private sector	Bride
K233	F	49	[40-50[	Analphabet	Household	Bride
K234	F	36	[30-40[	Secondary school	Private sector	Bachelor
K235	F	40	[40-50[	Secondary school	Civil servant	Bachelor
K236	M	81	≥60	Analphabet	Jobless	Bride
K237	F	61	≥60	Analphabet	Private sector	Widow(er)
K238	F	68	≥60	Analphabet	Household	Widow(er)
K239	F	20	[20-30[	Secondary school	Student	Bachelor
K240	M	80	≥60	Analphabet	Jobless	Bride
K241	F	56	[50-60[	Secondary school	Private sector	Bride
K242	F	39	[30-40[	Secondary school	Civil servant	Bride
K243	F	45	[40-50[	Higher education	Civil servant	Bachelor
K244	F	38	[30-40[	Secondary school	Private sector	Bachelor
K245	F	33	[30-40[	Primary school	Household	Bride
K246	F	76	≥60	Secondary school	Jobless	Widow(er)
K247	F	62	≥60	Analphabet	Household	Bride
K248	F	44	[40-50[	Secondary school	Private sector	Bachelor
K249	F	43	[40-50[	Secondary school	Private sector	Bride
K250	F	50	[50-60[	Secondary school	Household	Bride
K251	F	45	[40-50[	Secondary school	Household	Bachelor
K252	M	14	<20	Secondary school	Student	Bachelor
K253	F	43	[40-50[	Secondary school	Household	Bachelor
K254	F	61	≥60	Secondary school	Household	Bride
K255	M	71	≥60	Higher education	Jobless	Bride

K256	M	30	[30-40[	Secondary school	Civil servant	Bachelor
K257	M	61	≥60	Analphabet	Private sector	Bride
K258	F	44	[40-50[	Secondary school	Civil servant	Bachelor
K259	F	62	≥60	Analphabet	Household	Widow(er)
K260	F	46	[40-50[	Secondary school	Civil servant	Bachelor
K261	F	47	[40-50[	Primary school	Household	Bachelor
K262	M	47	[40-50[	Analphabet	Private sector	Bride
K263	F	35	[30-40[	Secondary school	Household	Bachelor
K264	M	45	[40-50[	Primary school	Private sector	Bachelor
K265	F	43	[40-50[	Secondary school	Private sector	Bride
K266	F	46	[40-50[	Secondary school	Private sector	Bride
K267	F	54	[50-60[	Primary school	Private sector	Bride
K268	F	69	≥60	Secondary school	Household	Bride
K269	F	71	≥60	Analphabet	Household	Widow(er)
K270	F	69	≥60	Analphabet	Household	Bride
K271	F	58	[50-60[	Secondary school	Household	Bachelor
K272	F	46	[40-50[	Secondary school	Private sector	Bride
K273	F	52	[50-60[	Secondary school	Private sector	Bachelor
K274	F	39	[30-40[	Secondary school	Household	Bachelor
K275	F	52	[50-60[	Primary school	Private sector	Bride
K276	F	48	[40-50[	Primary school	Private sector	Bachelor
K277	F	53	[50-60[	Secondary school	Household	Bride
K278	F	58	[50-60[	Primary school	Household	Widow(er)
K279	F	45	[40-50[	Secondary school	Private sector	Bride
K280	M	41	[40-50[	Higher education	Civil servant	Bride
K281	F	49	[40-50[	Secondary school	Civil servant	Bride
K282	F	53	[50-60[	Primary school	Private sector	Bachelor
K283	F	60	≥60	Analphabet	Private sector	Bride
K284	F	46	[40-50[	Secondary school	Household	Bachelor

K285	F	57	[50-60[	Analphabet	Household	Bachelor
K286	M	47	[40-50[	Higher education	Civil servant	Bachelor
K287	M	56	[50-60[	Analphabet	Private sector	Bride
K288	F	26	[20-30[	Higher education	Student	Bachelor
K289	M	28	[20-30[	Higher education	Student	Bachelor
K290	F	60	≥60	Analphabet	Private sector	Widow(er)
K291	F	32	[30-40[	Higher education	Private sector	Bachelor
K292	M	27	[20-30[	Higher education	Student	Bachelor
K293	M	42	[40-50[	Analphabet	Private sector	Bride
K294	F	41	[40-50[	Secondary school	Household	Bachelor
K295	M	28	[20-30[	Secondary school	Civil servant	Bachelor
K296	F	48	[40-50[	Primary school	Household	Bride
K297	F	68	≥60	Analphabet	Household	Bride
K298	F	64	≥60	Analphabet	Household	Bride
K299	M	59	[50-60[	Secondary school	Civil servant	Bride
K300	M	52	[50-60[	Secondary school	Civil servant	Bride
K301	M	25	[20-30[	Secondary school	Private sector	Bachelor
K302	F	75	≥60	Analphabet	Jobless	Widow(er)
K303	F	49	[40-50[	Secondary school	Household	Bachelor
K304	F	41	[40-50[	Primary school	Private sector	Bride
K305	F	63	≥60	Secondary school	Jobless	Widow(er)
K306	M	16	<20	Secondary school	Student	Bachelor
K307	F	50	[50-60[	Higher education	Civil servant	Bride
**Non-cancer patient**						
D001	M	45	[40-50[	Secondary school	Private sector	Bride
D002	F	64	≥60	Secondary school	Household	Bride
D003	F	52	[50-60[	Secondary school	Household	Bride

D004	M	42	[40-50[	Secondary school	Civil servant	Bride
D005	M	69	≥60	Analphabet	Jobless	Bride
D006	F	34	[30-40[	Secondary school	Household	Bride
D007	M	49	[40-50[	Higher education	Civil servant	Bride
D008	F	44	[40-50[	Secondary school	Household	Bachelor
D009	M	77	≥60	Analphabet	Jobless	Bride
D010	M	10	<20	Primary school	Student	Bachelor
D011	M	40	[40-50[	Secondary school	Private sector	Bride
D012	M	31	[30-40[	Higher education	Student	Bachelor
D013	F	38	[30-40[	Secondary school	Private sector	Bachelor
D014	F	55	[50-60[	Primary school	Household	Bride
D015	F	41	[40-50[	Secondary school	Private sector	Bachelor
D016	F	20	[20-30[	Secondary school	Student	Bachelor
D017	F	61	≥60	Secondary school	Household	Widow(er)
D018	F	23	[20-30[	Higher education	Student	Bachelor
D019	F	53	[50-60[	Secondary school	Private sector	Bride
D020	F	34	[30-40[	Secondary school	Jobless	Bride
D021	F	36	[30-40[	Higher education	Civil servant	Bride
D022	M	10	<20	Primary school	Student	Bachelor
D023	M	28	[20-30[	Secondary school	Civil servant	Bachelor
D024	F	22	[20-30[	Secondary school	Student	Bachelor
D025	F	38	[30-40[	Primary school	Household	Bachelor
D026	F	20	[20-30[	Secondary school	Student	Bachelor
D027	M	35	[30-40[	Secondary school	Private sector	Bride
D028	F	40	[40-50[	Higher education	Civil servant	Bride
D029	F	53	[50-60[	Primary school	Household	Bride
D030	F	45	[40-50[	Higher education	Civil servant	Bride
D031	F	36	[30-40[	Higher education	Household	Bachelor
D032	M	63	≥60	Primary school	Civil servant	Bride

D033	F	22	[20-30[	Secondary school	Student	Bachelor
D034	F	38	[30-40[	Higher education	Civil servant	Bride
D035	M	57	[50-60[	Secondary school	Private sector	Bride
D036	F	20	[20-30[	Higher education	Student	Bachelor
D037	F	54	[50-60[	Secondary school	Household	Bride
D038	F	27	[20-30[	Higher education	Student	Bachelor
D039	F	25	[20-30[	Secondary school	Household	Bride
D040	F	58	[50-60[	Secondary school	Household	Bride
D041	M	27	[20-30[	Higher education	Student	Bachelor
D042	F	66	≥60	Secondary school	Household	Bride
D043	M	10	<20	Primary school	Student	Bachelor
D044	F	37	[30-40[	Secondary school	Household	Bride
D045	F	78	≥60	Analphabet	Household	Widow(er)
D046	M	63	≥60	Secondary school	Private sector	Bride
D047	M	43	[40-50[	Primary school	Private sector	Bride
D048	F	63	≥60	Primary school	Household	Bride
D049	M	52	[50-60[	Secondary school	Civil servant	Bride
D050	F	23	[20-30[	Secondary school	Student	Bachelor
D051	M	42	[40-50[	Secondary school	Private sector	Bride
D052	F	23	[20-30[	Higher education	Student	Bachelor
D053	F	54	[50-60[	Secondary school	Household	Bride
D054	F	31	[30-40[	Secondary school	Household	Bachelor
D055	M	44	[40-50[	Secondary school	Civil servant	Bride
D056	M	43	[40-50[	Secondary school	Private sector	Bride
D057	M	40	[40-50[	Secondary school	Private sector	Bride
D058	M	47	[40-50[	Higher education	Civil servant	Bride
D059	M	10	<20	Primary school	Student	Bachelor
D060	M	41	[40-50[	Secondary school	Private sector	Bride
D061	M	37	[30-40[	Higher education	Civil servant	Bride

D062	F	17	<20	Secondary school	Student	Bachelor
D063	F	30	[30-40[	Secondary school	Household	Bachelor
D064	F	38	[30-40[	Higher education	Private sector	Bachelor
D065	M	30	[30-40[	Secondary school	Private sector	Bachelor
D066	F	33	[30-40[	Secondary school	Household	Bride
D067	F	84	≥60	Analphabet	Household	Widow(er)
D068	F	38	[30-40[	Secondary school	Private sector	Bachelor
D069	M	48	[40-50[	Higher education	Private sector	Bride
D070	F	39	[30-40[	Primary school	Household	Bride
D071	F	44	[40-50[	Higher education	Private sector	Bride
D072	F	34	[30-40[	Secondary school	Civil servant	Bride
D073	F	10	<20	Primary school	Student	Bachelor
D074	F	42	[40-50[	Secondary school	Private sector	Bride
D075	F	36	[30-40[	Higher education	Private sector	Bride
D076	M	29	[20-30[	Higher education	Student	Bachelor
D077	F	20	[20-30[	Higher education	Student	Bachelor
D078	M	29	[20-30[	Secondary school	Private sector	Bachelor
D079	F	10	<20	Primary school	Student	Bachelor
D080	M	81	≥60	Analphabet	Jobless	Bride
D081	F	29	[20-30[	Secondary school	Private sector	Bride
D082	F	51	[50-60[	Secondary school	Household	Widow(er)
D083	M	35	[30-40[	Secondary school	Civil servant	Bride
D084	F	51	[50-60[	Secondary school	Household	Bride
D085	F	42	[40-50[	Secondary school	Household	Bride
D086	M	64	≥60	Secondary school	Civil servant	Bride
D087	M	56	[50-60[	Secondary school	Jobless	Bride
D088	F	33	[30-40[	Secondary school	Household	Bride
D089	M	34	[30-40[	Secondary school	Private sector	Bride
D090	M	58	[50-60[	Secondary school	Private sector	Bride

D091	M	54	[50-60[	Secondary school	Civil servant	Bride
D092	M	49	[40-50[	Secondary school	Private sector	Bride
D093	M	48	[40-50[	Secondary school	Private sector	Bride
D094	M	52	[50-60[	Secondary school	Private sector	Bride
D095	F	34	[30-40[	Secondary school	Household	Bride
D096	F	29	[20-30[	Higher education	Private sector	Bachelor
D097	M	25	[20-30[	Higher education	Student	Bachelor
D098	F	55	[50-60[	Secondary school	Civil servant	Bride
D099	M	41	[40-50[	Primary school	Private sector	Bachelor
D100	F	48	[40-50[	Secondary school	Household	Bachelor
D101	F	42	[40-50[	Secondary school	Household	Bride
D102	F	45	[40-50[	Secondary school	Private sector	Bride
D103	F	23	[20-30[	Secondary school	Student	Bachelor
D104	M	47	[40-50[	Secondary school	Civil servant	Bride
D105	M	15	<20	Secondary school	Student	Bachelor
D106	F	68	≥60	Primary school	Jobless	Bachelor
D107	M	39	[30-40[	Secondary school	Private sector	Bride
D108	M	47	[40-50[	Secondary school	Private sector	Bride
D109	F	36	[30-40[	Secondary school	Private sector	Bachelor
D110	M	45	[40-50[	Secondary school	Civil servant	Bride
D111	F	42	[40-50[	Secondary school	Private sector	Widow(er)
D112	F	20	[20-30[	Secondary school	Civil servant	Bride
D113	F	23	[20-30[	Secondary school	Private sector	Bride
D114	M	47	[40-50[	Primary school	Private sector	Bride
D115	M	20	[20-30[	Higher education	Private sector	Bride
D116	F	68	≥60	Secondary school	Civil servant	Bride
D117	M	39	[30-40[	Primary school	Private sector	Bride
D118	M	47	[40-50[	Secondary school	Civil servant	Bride
D119	F	36	[30-40[	Higher education	Private sector	Bride

D120	M	32	[30-40[	Secondary school	Civil servant	Bachelor
D121	F	80	≥60	Secondary school	Private sector	Bachelor
D122	F	53	[50-60[	Primary school	Private sector	Bride
D123	F	29	[20-30[	Secondary school	Private sector	Bride
D124	M	66	≥60	Secondary school	Private sector	Bride
D125	M	71	≥60	Secondary school	Private sector	Bride
D126	M	42	[40-50[	Secondary school	Private sector	Bachelor
D127	F	28	[20-30[	Secondary school	Private sector	Bachelor
D128	M	33	[30-40[	Secondary school	Private sector	Bride
D129	F	72	≥60	Primary school	Private sector	Bachelor
D130	F	60	≥60	Secondary school	Private sector	Widow(er)
D131	F	70	≥60	Secondary school	Civil servant	Bride
D132	M	29	[20-30[	Secondary school	Private sector	Bride
D133	F	35	[30-40[	Secondary school	Private sector	Bride
D134	M	46	[40-50[	Analphabet	Private sector	Bachelor
D135	M	39	[30-40[	Secondary school	Private sector	Widow(er)
D136	F	65	≥60	Secondary school	Private sector	Bride
D137	M	44	[40-50[	Primary school	Private sector	Bride
D138	F	65	≥60	Analphabet	Private sector	Bride
D139	F	44	[40-50[	Secondary school	Private sector	Widow(er)
D140	F	64	≥60	Secondary school	Civil servant	Bride
D141	F	56	[50-60[	Secondary school	Civil servant	Bride
D142	F	66	≥60	Analphabet	Private sector	Bride
D143	M	34	[30-40[	Primary school	Private sector	Widow(er)
D144	F	58	[50-60[	Primary school	Private sector	Widow(er)
D145	F	54	[50-60[	Secondary school	Private sector	Bachelor
D146	F	48	[40-50[	Higher education	Civil servant	Bachelor
D147	M	52	[50-60[	Secondary school	Civil servant	Bride
D148	M	34	[30-40[	Secondary school	Jobless	Bride

D149	F	29	[20-30[	Higher education	Jobless	Bachelor
D150	F	38	[30-40[	Secondary school	Jobless	Bride
D151	F	36	[30-40[	Higher education	Civil servant	Bride
D152	M	10	<20	Primary school	Student	Bachelor
D153	M	28	[20-30[	Secondary school	Civil servant	Bachelor
D154	F	22	[20-30[	Secondary school	Student	Bachelor
D155	F	38	[30-40[	Primary school	Household	Bachelor
D156	F	20	[20-30[	Secondary school	Student	Bachelor
D157	M	35	[30-40[	Secondary school	Private sector	Bride
D158	F	40	[40-50[	Higher education	Civil servant	Bride
D159	F	53	[50-60[	Primary school	Household	Bride
D160	F	45	[40-50[	Higher education	Civil servant	Bride
D161	F	36	[30-40[	Higher education	Household	Bachelor
D162	M	63	≥60	Primary school	Civil servant	Bride
D163	F	22	[20-30[	Secondary school	Student	Bachelor
D164	F	38	[30-40[	Higher education	Civil servant	Bride
D165	M	57	[50-60[	Secondary school	Private sector	Bride
D166	F	20	[20-30[	Higher education	Student	Bachelor
D167	F	54	[50-60[	Secondary school	Household	Bride
D168	F	27	[20-30[	Higher education	Student	Bachelor
D169	F	25	[20-30[	Secondary school	Household	Bride
D170	F	58	[50-60[	Secondary school	Household	Bride
D171	M	27	[20-30[	Higher education	Student	Bachelor
D172	F	66	≥60	Secondary school	Household	Bride
D173	M	10	<20	Primary school	Student	Bachelor
D174	F	37	[30-40[	Secondary school	Household	Bride
D175	F	78	≥60	Analphabet	Household	Widow(er)
D176	M	63	≥60	Secondary school	Private sector	Bride
D177	M	43	[40-50[	Primary school	Private sector	Bride

D178	F	63	≥60	Primary school	Household	Bride
D179	M	52	[50-60[	Secondary school	Civil servant	Bride
D180	F	23	[20-30[	Secondary school	Student	Bachelor
D181	M	42	[40-50[	Secondary school	Private sector	Bride
D182	F	23	[20-30[	Higher education	Student	Bachelor
D183	F	54	[50-60[	Secondary school	Household	Bride
D184	F	31	[30-40[	Secondary school	Household	Bachelor
D185	M	44	[40-50[	Secondary school	Civil servant	Bride
D186	M	43	[40-50[	Secondary school	Private sector	Bride
D187	M	40	[40-50[	Secondary school	Private sector	Bride
D188	M	47	[40-50[	Higher education	Civil servant	Bride
D189	M	10	<20	Primary school	Student	Bachelor
D190	M	41	[40-50[	Secondary school	Private sector	Bride
D191	M	37	[30-40[	Higher education	Civil servant	Bride
D192	F	17	<20	Secondary school	Student	Bachelor
D193	F	30	[30-40[	Secondary school	Household	Bachelor
D194	F	38	[30-40[	Higher education	Private sector	Bachelor
D195	M	30	[30-40[	Secondary school	Private sector	Bachelor
D196	F	33	[30-40[	Secondary school	Household	Bride
D197	F	84	≥60	Analphabet	Household	Widow(er)
D198	F	38	[30-40[	Secondary school	Private sector	Bachelor
D199	M	48	[40-50[	Higher education	Private sector	Bride
D200	F	39	[30-40[	Primary school	Household	Bride
Legend: M, male; F, female; code with K, cancer patient; code with D, non-cancer patient						

S2. Patients and cancer treatments

Patients	Cancer, features, and treatment status															
**Patient Code**	**Cancer status**	**Location of the pathology**	**Cancer stage**	**Classification**	**From when?**	**Chemo-therapy?**	**Number of chemotherapy sessions**	**Type of Chemotherapy**	**Diarr-hea**	**Abdo-minal pain**	**Consti-pation**	**Vomi-ting**	**Head-ache**	**Nau-sea**	**Fever**	**Asthenia**
K001	Yes	Cavum	Stage 3	Lymph nodes	6 Months	No			No	Yes	Yes	No	Yes	Yes	Yes	Yes
K002	Yes	Prostate	Stage 4	Metastasis	>12 Months	No			No	Yes	Yes	Yes	No	Yes	No	Yes
K003	Yes	Breast	Stage 4	Metastasis	12 Months	Yes	More than 6 times	Adjuvant	Yes	Yes	No	No	No	No	Yes	Yes
K004	Yes	Pancreas	Stage 4	Metastasis	>12 Months	Yes	Less than 4 times	neo-Adjuvant	Yes	Yes	No	No	No	Yes	Yes	Yes
K005	Yes	Colorectal	Stage 4	Metastasis	>12 Months	Yes	More than 6 times	Adjuvant	Yes	Yes	No	No	Yes	No	Yes	Yes
K006	Yes	Liver	Stage 4	Metastasis	12 Months	No			No	Yes	Yes	No	Yes	Yes	Yes	Yes
K007	Yes	Cervical	Stage 3	Lymph nodes	6 Months	No			No	Yes	Yes	Yes	No	Yes	No	No
K008	Yes	Kaposi sarcoma	Unknwon stage		12 Months	No			No	Yes	Yes	No	Yes	No	No	Yes
K009	Yes	Breast	Stage 3	Lymph nodes	12 Months	Yes	Less than 4 times	Exclusive	No	Yes	Yes	No	No	No	No	No
K010	Yes	Leukemia	Stage 4	Metastasis	12 Months	No			No	Yes	Yes	No	No	Yes	No	No
K011	Yes	Breast	Stage 3	Lymph nodes	12 Months	No			Yes	Yes	Yes	No	Yes	No	No	Yes
K012	Yes	Lung	Stage 4	Metastasis	>12 Months	Yes	Less than 4 times	Palliative	No	Yes	Yes	No	No	Yes	Yes	Yes
K013	Yes	Breast	Stage 4	Metastasis	>12 Months	Yes	Less than 4 times	Exclusive	Yes	Yes	No	No	No	No	Yes	Yes
K014	Yes	Osteosarcoma	Stage 4	Metastasis	6 Months	No			No	Yes	Yes	No	No	Yes	Yes	Yes
K015	Yes	Breast	Stage 3	Lymph nodes	12 Months	Yes	More than 6 times	Exclusive	No	No	Yes	No	No	No	No	Yes
K016	Yes	Liver	Unknwon stage		6 Months	No			No	Yes	Yes	No	No	Yes	No	Yes
K017	Yes	Osteosarcoma	Stage 4	Metastasis	>12 Months	Yes	Less than 4 times	Exclusive	No	Yes	Yes	No	No	No	No	Yes
K018	Yes	Lung	Stage 4	Metastasis	12 Months	Yes	More than 6 times	Palliative	No	No	Yes	No	Yes	Yes	No	Yes
K019	Yes	Cholangiocarcinoma	Stage 4	Metastasis	6 Months	No			No	Yes	Yes	No	No	Yes	Yes	Yes
K020	Yes	Cavum	Stage 3	Lymph nodes	6 Months	Yes	Less than 4 times	Adjuvant	No	No	Yes	No	No	No	No	No

K021	Yes	Liver	Stage 3	Lymph nodes	6 Months	No			No	Yes	Yes	No	No	No	Yes	No
K022	Yes	Colorectal	Stage 3	Lymph nodes	6 Months	Yes	Less than 4 times	neo-Adjuvant	No	Yes	Yes	No	No	No	Yes	No
K023	Yes	Lung	Stage 3	Metastasis	12 Months	Yes	Less than 4 times	Exclusive	Yes	Yes	Yes	Yes	Yes	Yes	Yes	Yes
K024	Yes	Liver	Unknwon stage		6 Months	No			Yes	Yes	No	No	No	Yes	Yes	Yes
K025	Yes	Leukemia	Unknwon stage		6 Months	No			No	No	No	No	No	No	No	Yes
K026	Yes	Leukemia	Unknwon stage		6 Months	Yes	Less than 4 times	Exclusive	No	Yes	Yes	No	No	Yes	Yes	Yes
K027	Yes	Prostate	Stage 3	Lymph nodes	12 Months	No			No	Yes	Yes	No	No	Yes	Yes	Yes
K028	Yes	Colorectal	Stage 3	Lymph nodes	6 Months	No			No	Yes	Yes	No	Yes	Yes	No	Yes
K029	Yes	Liver	Stage 3	Lymph nodes	6 Months	No			Yes	Yes	No	Yes	Yes	Yes	Yes	Yes
K030	Yes	Leukemia	Unknwon stage		6 Months	No			No	No	Yes	No	No	No	Yes	Yes
K031	Yes	Pancreas	Stage 3	Lymph nodes	12 Months	No			No	Yes	Yes	No	No	Yes	Yes	Yes
K032	Yes	Breast	Stage 4	Metastasis	>12 Months	No			No	Yes	Yes	No	Yes	Yes	Yes	Yes
K033	Yes	Breast	Stage 4	Metastasis	6 Months	No			No	Yes	Yes	No	No	Yes	No	Yes
K034	Yes	Pancreas	Stage 3	Lymph nodes	12 Months	No			No	Yes	No	No	No	No	Yes	Yes
K035	Yes	Osteosarcoma	Stage 3	Lymph nodes	12 Months	Yes	Less than 4 times	Adjuvant	No	No	Yes	No	No	No	No	Yes
K036	Yes	Breast	Stage 4	Metastasis	>12 Months	Yes	Less than 4 times	Adjuvant	No	Yes	Yes	No	Yes	Yes	Yes	Yes
K037	Yes	Breast	Stage 3	Lymph nodes	>12 Months	Yes	More than 6 times	neo-Adjuvant	Yes	Yes	Yes	Yes	No	Yes	No	Yes
K038	Yes	Cholangiocarcinoma	Stage 4	Metastasis	>12 Months	Yes	More than 6 times	Palliative	No	No	Yes	Yes	No	Yes	Yes	Yes
K039	Yes	Prostate	Stage 4	Metastasis	>12 Months	No			Yes	Yes	No	No	No	No	Yes	Yes
K040	Yes	Prostate	Stage 4	Metastasis	>12 Months	No			No	Yes	Yes	No	No	No	No	Yes
K041	Yes	Prostate	Stage 4	Metastasis	>12 Months	No			No	Yes	Yes	No	No	No	No	Yes
K042	Yes	Cervical	Unknwon stage		6 Months	No			Yes	Yes	No	No	No	No	Yes	Yes
K043	Yes	Cervical	Stage 3	Lymph nodes	12 Months	No			No	Yes	Yes	No	No	No	No	No
K044	Yes	Breast	Stage 4	Metastasis	>12 Months	No			No	Yes	Yes	No	No	No	Yes	Yes
K045	Yes	Osteosarcoma	Stage 4	Metastasis	6 Months	No			No	Yes	Yes	No	No	No	Yes	Yes
K046	Yes	Cervical	Stage 3	Lymph nodes	>12 Months	Yes	More than 6 times	Exclusive	No	Yes	Yes	No	No	No	No	No
K047	Yes	Kaposi sarcoma	Unknwon stage		12 Months	Yes	Less than 4 times	Exclusive	No	Yes	Yes	No	No	No	No	No

K048	Yes	Breast	Stage 3	Lymph nodes	>12 Months	Yes	More than 6 times	Adjuvant	No	No	Yes	No	No	Yes	No	No
K049	Yes	Breast	Stage 4	Metastasis	>12 Months	No			No	Yes	Yes	No	No	Yes	Yes	Yes
K050	Yes	Leukemia	Stage 4	Metastasis	>12 Months	No			No	Yes	Yes	No	No	No	No	Yes
K051	Yes	Cholangiocarcinoma	Stage 4	Metastasis	6 Months	No			No	Yes	Yes	No	No	No	No	Yes
K052	Yes	Breast	Stage 4	Metastasis	>12 Months	No			No	No	No	No	No	No	No	No
K053	Yes	Cervical	Stage 3	Lymph nodes	6 Months	No			No	Yes	Yes	No	No	Yes	Yes	Yes
K054	Yes	Cervical	Stage 4	Metastasis	>12 Months	No			No	Yes	Yes	No	No	No	Yes	Yes
K055	Yes	Osteosarcoma	Stage 4	Metastasis	>12 Months	Yes		Adjuvant	No	Yes	Yes	No	No	Yes	Yes	Yes
K056	Yes	Breast	Stage 4	Metastasis	>12 Months	Yes		Adjuvant	No	No	Yes	No	Yes	Yes	Yes	Yes
K057	Yes	Lung	Stage 4	Metastasis	12 Months	Yes	Less than 4 times	Exclusive	No	No	Yes	Yes	Yes	Yes	Yes	Yes
K058	Yes	Breast	Stage 4	Metastasis	>12 Months	Yes	More than 6 times	Palliative	No	Yes	Yes	No	No	No	Yes	Yes
K059	Yes	Colorectal	Stage 3	Lymph nodes	6 Months	No			No	Yes	Yes	No	No	Yes	Yes	Yes
K060	Yes	Breast	Stage 4	Metastasis	12 Months	Yes	More than 6 times	Adjuvant	Yes	Yes	No	No	No	Yes	Yes	Yes
K061	Yes	Breast	Stage 4	Metastasis	12 Months	No			No	Yes	Yes	No	Yes	Yes	Yes	Yes
K062	Yes	Stomach	Stage 3	Lymph nodes	12 Months	No			No	Yes	Yes	Yes	Yes	Yes	Yes	Yes
K063	Yes	Leukemia	Stage 4	Metastasis	>12 Months	Yes	More than 6 times	Exclusive	No	No	No	No	No	No	No	Yes
K064	Yes	Cervical	Stage 4	Metastasis	>12 Months	Yes	More than 6 times	Exclusive	No	Yes	Yes	No	No	Yes	Yes	Yes
K065	Yes	Prostate	Unknwon stage		12 Months	No			No	Yes	Yes	Yes	Yes	Yes	Yes	Yes
K066	Yes	Leukemia	Stage 3	Lymph nodes	12 Months	Yes	More than 6 times	Exclusive	No	No	Yes	No	No	No	No	No
K067	Yes	Liver	Stage 4	Metastasis	>12 Months	No			No	Yes	Yes	Yes	Yes	Yes	Yes	Yes
K068	Yes	Colorectal	Stage 3	Lymph nodes	12 Months	Yes	More than 6 times	neo-Adjuvant	No	Yes	Yes	Yes	Yes	Yes	No	Yes
K069	Yes	Liver	Stage 4	Metastasis	12 Months	No			No	Yes	Yes	No	No	No	No	Yes
K070	Yes	Liver	Stage 4	Metastasis	12 Months	No			Yes	No	No	Yes	No	No	Yes	Yes
K071	Yes	Osteosarcoma	Stage 4	Metastasis	12 Months	No			No	Yes	Yes	No	No	No	No	Yes
K072	Yes	Cervical	Stage 4	Metastasis	12 Months	No			No	Yes	Yes	No	No	No	Yes	Yes
K073	Yes	Leukemia	Stage 4	Metastasis	12 Months	No			No	Yes	Yes	No	No	No	No	Yes
K074	Yes	Colorectal	Stage 4	Metastasis	12 Months	No			No	Yes	Yes	No	Yes	No	Yes	Yes

K075	Yes	Breast	Stage 4	Metastasis	>12 Months	Yes	More than 6 times	Adjuvant	Yes	Yes	Yes	No	No	Yes	No	Yes
K076	Yes	Breast	Stage 4	Metastasis	>12 Months	No	Less than 4 times	Adjuvant	No	Yes	Yes	No	Yes	Yes	Yes	Yes
K077	Yes	Kaposi sarcoma	Stage 4	Metastasis	12 Months	Yes	More than 6 times	Exclusive	Yes	Yes	No	No	No	Yes	Yes	Yes
K078	Yes	Stomach	Stage 3	Lymph nodes	12 Months	No			No	Yes	Yes	Yes	Yes	Yes	Yes	Yes
K079	Yes	Breast	Stage 4	Metastasis	>12 Months	Yes	Less than 4 times	neo-Adjuvant	No	Yes	Yes	No	No	No	No	No
K080	Yes	Cervical	Unknwon stage		6 Months	Yes	Less than 4 times	Exclusive	No	No	Yes	No	No	No	No	No
K081	Yes	Cervical	Stage 4	Metastasis	>12 Months	No			Yes	No	No	No	No	No	No	Yes
K082	Yes	Colorectal	Stage 3	Lymph nodes	>12 Months	No			No	No	Yes	No	No	No	No	Yes
K083	Yes	Breast	Stage 3	Lymph nodes	>12 Months	Yes	More than 6 times	neo-Adjuvant	No	Yes	Yes	No	No	No	Yes	Yes
K084	Yes	Pancreas	Unknwon stage		6 Months	Yes	Less than 4 times	neo-Adjuvant	No	Yes	Yes	No	No	No	No	Yes
K085	Yes	Breast	Stage 4	Metastasis	>12 Months	No			No	Yes	Yes	No	No	Yes	No	No
K086	Yes	Prostate	Stage 4	Metastasis	>12 Months	No			No	Yes	Yes	No	No	No	Yes	Yes
K087	Yes	Leukemia	Stage 3	Lymph nodes	12 Months	Yes	Less than 4 times	Exclusive	No	No	No	No	No	No	No	No
K088	Yes	Breast	Stage 3	Lymph nodes	>12 Months	Yes	More than 6 times	Adjuvant	No	No	No	No	No	No	No	No
K089	Yes	Prostate	Stage 4	Metastasis	>12 Months	No			No	Yes	Yes	No	No	No	No	Yes
K090	Yes	Leukemia	Unknwon stage		>12 Months	No			No	Yes	Yes	No	No	Yes	Yes	Yes
K091	Yes	Colorectal	Stage 3	Lymph nodes	12 Months	No			Yes	Yes	No	No	No	Yes	Yes	Yes
K092	Yes	Breast	Stage 3	Lymph nodes	>12 Months	No			No	Yes	No	No	No	No	No	No
K093	Yes	Cervical	Stage 3	Lymph nodes	12 Months	Yes	Less than 4 times	neo-Adjuvant	No	Yes	Yes	No	No	No	No	No
K094	Yes	Cavum	Unknwon stage		6 Months	No			No	No	Yes	No	No	No	No	No
K095	Yes	Cervical	Unknwon stage		12 Months	Yes	More than 6 times	Adjuvant	No	Yes	Yes	No	No	No	Yes	Yes
K096	Yes	Leukemia	Stage 3	Lymph nodes	12 Months	Yes	More than 6 times	Exclusive	Yes	Yes	No	No	No	No	No	Yes
K097	Yes	Breast	Stage 3	Lymph nodes	12 Months	Yes	Less than 4 times	neo-Adjuvant	No	No	No	No	No	No	No	No
K098	Yes	Leukemia	Unknwon stage		12 Months	Yes	More than 6 times	Exclusive	Yes	Yes	No	No	Yes	Yes	Yes	Yes
K099	Yes	Leukemia	Stage 4	Metastasis	>12 Months	Yes	Less than 4 times	Exclusive	Yes	Yes	No	No	Yes	Yes	Yes	Yes

K100	Yes	Cervical	Stage 4	Metastasis	12 Months	Yes	More than 6 times	Palliative	No	No	Yes	No	No	No	Yes	Yes
K101	Yes	Kaposi sarcoma	Unknwon stage		12 Months	Yes	Less than 4 times	Exclusive	No	Yes	Yes	No	No	No	No	No
K102	Yes	Cholangiocarcinoma	Unknwon stage		6 Months	Yes	More than 6 times	Exclusive	Yes	No	No	No	No	No	No	Yes
K103	Yes	Colorectal	Stage 2	Lymph nodes	12 Months	Yes	Less than 4 times	neo-Adjuvant	No	No	Yes	No	No	No	No	Yes
K104	Yes	Colorectal	Stage 4	Metastasis	>12 Months	No			Yes	Yes	Yes	No	No	No	No	Yes
K105	Yes	Breast	Stage 4	Metastasis	>12 Months	Yes	More than 6 times	neo-Adjuvant	No	No	No	No	No	No	No	No
K106	Yes	Breast	Stage 4	Metastasis	>12 Months	Yes	More than 6 times	Palliative	No	Yes	Yes	No	Yes	No	No	Yes
K107	Yes	Leukemia	Unknwon stage		12 Months	Yes	More than 6 times	Palliative	No	Yes	No	No	No	No	No	No
K108	Yes	Colorectal	Stage 3	Lymph nodes	12 Months	Yes	Less than 4 times	Adjuvant	No	Yes	No	No	No	No	No	No
K109	Yes	Breast	Stage 4	Metastasis	>12 Months	Yes	More than 6 times	Adjuvant	No	No	Yes	Yes	Yes	Yes	Yes	Yes
K110	Yes	Breast	Stage 3	Lymph nodes	12 Months	Yes	Less than 4 times	neo-Adjuvant	Yes	Yes	No	No	No	No	No	Yes
K111	Yes	Osteosarcoma	Unknwon stage		12 Months	Yes	More than 6 times	Exclusive	Yes	No	nn	No	No	No	No	No
K112	Yes	Breast	Stage 3	Lymph nodes	12 Months	Yes	Less than 4 times	neo-Adjuvant	No	Yes	Yes	No	Yes	Yes	Yes	Yes
K113	Yes	Breast	Stage 3	Lymph nodes		Yes	Less than 4 times	Exclusive	No	Yes	Yes	No	No	No	No	No
K114	Yes	Breast	Stage 3	Lymph nodes	12 Months	Yes	Less than 4 times	Adjuvant	No	Yes	Yes	No	No	No	No	No
K115	Yes	Cervical	Stage 3	Lymph nodes	12 Months	Yes	Less than 4 times	Exclusive	No	Yes	Yes	No	No	No	No	No
K116	Yes	Breast	Stage 3	Lymph nodes	12 Months	Yes	Less than 4 times	neo-Adjuvant	No	No	Yes	Yes	Yes	Yes	Yes	Yes
K117	Yes	Stomach	Unknwon stage		12 Months	Yes	Less than 4 times	neo-Adjuvant	Yes	No	No	No	No	No	Yes	Yes
K118	Yes	Prostate	Stage 4	Metastasis	>12 Months	Yes	More than 6 times	Exclusive	No	Yes	Yes	No	No	No	No	No
K119	Yes	Cervical	Stage 3	Lymph nodes	>12 Months	Yes	More than 6 times	Adjuvant	Yes	Yes	No	Yes	Yes	No	Yes	Yes
K120	Yes	Stomach	Stage 4	Metastasis	>12 Months	Yes	More than 6 times	Exclusive	Yes	Yes	No	Yes	Yes	No	Yes	Yes
K121	Yes	Breast	Stage 3	Lymph nodes	12 Months	Yes	More than 6 times	neo-Adjuvant	Yes	Yes	No	Yes	Yes	Yes	Yes	Yes
K122	Yes	Leukemia	Unknwon stage		>12 Months	Yes	Less than 4 times	Exclusive	No	No	Yes	No	No	No	No	Yes
K123	Yes	Breast	Stage 3	Lymph nodes	12 Months	Yes	Less than 4 times	Adjuvant	No	Yes	Yes	No	No	No	No	No

K124	Yes	Stomach	Unknwon stage		12 Months	Yes	Less than 4 times	Adjuvant	No	No	Yes	No	No	No	No	No
K125	Yes	Cervical	Stage 3	Lymph nodes	12 Months	Yes	Less than 4 times	Exclusive	No	No	No	No	No	No	No	No
K126	Yes	Breast	Stage 4	Metastasis	>12 Months	Yes	Less than 4 times	neo-Adjuvant	No	Yes	Yes	No	No	No	No	No
K127	Yes	Breast	Unknwon stage		>12 Months	Yes	More than 6 times	neo-Adjuvant	No	Yes	Yes	No	Yes	No	Yes	No
K128	Yes	Breast	Stage 3	Lymph nodes	>12 Months	Yes	Less than 4 times	neo-Adjuvant	No	Yes	Yes	No	No	No	No	No
K129	Yes	Breast	Stage 3	Lymph nodes	>12 Months	Yes	Less than 4 times	neo-Adjuvant	Yes	Yes	Yes	No	No	No	Yes	Yes
K130	Yes	Cervical	Stage 4	Metastasis	6 Months	No			No	Yes	Yes	No	Yes	Yes	Yes	Yes
K131	Yes	Breast	Stage 4	Metastasis	>12 Months	No			No	No	Yes	No	No	No	No	Yes
K132	Yes	Cervical	Stage 4	Metastasis	>12 Months	No			No	Yes	Yes	Yes	Yes	Yes	Yes	Yes
K133	Yes	Breast	Stage 3	Lymph nodes	6 Months	No			Yes	Yes	No	Yes	Yes	Yes	Yes	Yes
K134	Yes	Cholangiocarcinoma	Stage 4	Metastasis	6 Months	No			No	No	Yes	No	No	Yes	No	Yes
K135	Yes	Stomach	Stage 4	Metastasis	>12 Months	No			Yes	No	No	No	Yes	No	Yes	No
K136	Yes	Colorectal	Stage 4	Metastasis	>12 Months	No			No	Yes	Yes	No	Yes	Yes	Yes	Yes
K137	Yes	Leukemia	Stage 4	Metastasis	6 Months	Yes	Less than 4 times	Exclusive	Yes	Yes	No	No	No	No	No	Yes
K138	Yes	Stomach	Stage 3	Lymph nodes	12 Months	Yes	More than times	Exclusive	No	Yes	Yes	No	No	No	No	No
K139	Yes	Leukemia	Stage 4	Metastasis	12 Months	Yes	More than 6 times	Exclusive	Yes	Yes	No	Yes	Yes	Yes	Yes	Yes
K140	Yes	Pancreas	Stage 4	Metastasis	>12 Months	Yes	Less than 4 times	Palliative	No	Yes	Yes	No	No	No	No	No
K141	Yes	Kaposi sarcoma	Stage 3	Lymph nodes	12 Months	Yes	More than 5 times	Exclusive	No	No	Yes	No	No	nn	No	No
K142	Yes	Cavum	Stage 4	Metastasis	>12 Months	Yes	More than 6 times	Exclusive	Yes	No	No	Yes	Yes	Yes	Yes	Yes
K143	Yes	Osteosarcoma	Stage 4	Metastasis	>12 Months	Yes	Less than 4 times	Exclusive	Yes	No	No	No	No	No	No	Yes
K144	Yes	Kaposi sarcoma	Unknwon stage		6 Months	Yes	Less than 4 times	Exclusive	No	Yes	Yes	No	No	No	Yes	Yes
K145	Yes	Breast	Stage 4	Metastasis	>12 Months	Yes	More than 6 times	Adjuvant	No	Yes	Yes	No	No	Yes	Yes	Yes
K146	Yes	Cervical	Stage 4	Metastasis	6 Months	No			No	Yes	Yes	No	No	No	Yes	Yes
K147	Yes	Cavum	Stage 4	Metastasis	>12 Months	Yes	More than 6 times	Exclusive	Yes	Yes	No	Yes	Yes	Yes	Yes	Yes
K148	Yes	Kaposi sarcoma	Unknwon stage		12 Months	Yes	Less than 4 times		No	Yes	Yes	No	No	No	No	No

K149	Yes	Breast	Stage 4	Metastasis	>12 Months	Yes	More than 6 times	Adjuvant	No	Yes	Yes	Yes	Yes	Yes	Yes	Yes
K150	Yes	Breast	Stage 4	Metastasis	>12 Months	Yes	More than 6 times	Adjuvant	No	Yes	Yes	Yes	Yes	Yes	Yes	Yes
K151	Yes	Cholangiocarcinoma	Stage 4	Metastasis	>12 Months	No			No	Yes	Yes	Yes	Yes	Yes	Yes	Yes
K152	Yes	Breast	Stage 4	Metastasis	>12 Months	Yes	More than 6 times	Adjuvant	Yes	No	No	Yes	Yes	Yes	Yes	Yes
K153	Yes	Breast	Stage 3	Lymph nodes	6 Months	No			No	No	No	No	No	No	No	No
K154	Yes	Breast	Stage 4	Metastasis	>12 Months	Yes	More than 6 times	Adjuvant	No	Yes	Yes	No	No	No	No	Yes
K155	Yes	Pancreas	Stage 4	Metastasis	>12 Months	Yes	More than 6 times	Exclusive	No	Yes	Yes	No	No	No	Yes	Yes
K156	Yes	Breast	Stage 4	Metastasis	>12 Months	Yes	Less than 4 times	Palliative	No	Yes	Yes	No	No	No	No	Yes
K157	Yes	Breast	Stage 4	Metastasis	>12 Months	Yes	More than 6 times	Palliative	No	Yes	Yes	No	No	No	Yes	Yes
K158	Yes	Leukemia	Stage 4	Metastasis	>12 Months	Yes	Less than 4 times	Exclusive	No	Yes	Yes	No	No	No	No	Yes
K159	Yes	Cavum	Unknwon stage		>12 Months	Yes	More than 6 times	Exclusive	No	Yes	Yes	No	No	No	No	No
K160	Yes	Breast	Stage 4	Metastasis	>12 Months	Yes	More than 6 times	neo-Adjuvant	Yes	Yes	No	No	No	No	No	No
K161	Yes	Cavum	Unknwon stage		12 Months	Yes	More than 6 times	Exclusive	No	No	No	No	No	No	No	Yes
K162	Yes	Cervical	Stage 4	Metastasis	12 Months	Yes	More than 6 times	Exclusive	No	Yes	Yes	Yes	Yes	Yes	No	Yes
K163	Yes	Breast	Stage 3	Lymph nodes	12 Months	Yes	Less than 4 times	Adjuvant	No	Yes	Yes	No	No	No	No	No
K164	Yes	Cervical	Stage 4	Metastasis	>12 Months	Yes	More than 6 times	neo-Adjuvant	Yes	Yes	No	Yes	Yes	Yes	Yes	Yes
K165	Yes	Cavum	Stage 3	Lymph nodes	12 Months	Yes	More than 6 times	Exclusive	No	Yes	Yes	No	Yes	Yes	Yes	Yes
K166	Yes	Prostate	Stage 4	Metastasis	>12 Months	Yes	More than 6 times	Palliative	No	Yes	Yes	No	No	Yes	Yes	Yes
K167	Yes	Pancreas	Stage 4	Metastasis	>12 Months	No			No	Yes	Yes	Yes	No	Yes	Yes	Yes
K168	Yes	Stomach	Unknwon stage		12 Months	Yes	Less than 4 times	neo-Adjuvant	Yes	No	No	Yes	No	Yes	No	Yes
K169	Yes	Stomach	Stage 4	Metastasis	>12 Months	Yes	Less than 4 times	neo-Adjuvant	No	Yes	Yes	Yes	No	Yes	Yes	Yes
K170	Yes	Breast	Stage 4	Metastasis	>12 Months	Yes	More than 6 times	Palliative	No	Yes	Yes	No	No	No	Yes	Yes
K171	Yes	Cholangiocarcinoma	Stage 4	Metastasis	>12 Months	No			No	Yes	Yes	No	No	No	No	Yes
K172	Yes	Colorectal	Stage 4	Metastasis	>12 Months	No			Yes	Yes	No	No	Yes	Yes	Yes	Yes

K173	Yes	Osteosarcoma	Stage 3	Metastasis	>12 Months	Yes	More than 6 times	Exclusive	No	Yes	Yes	No	No	No	No	No
K174	Yes	Colorectal	Stage 4	Metastasis	>12 Months	Yes	More than 6 times	Palliative	No	Yes	Yes	Yes	Yes	Yes	Yes	Yes
K175	Yes	Kaposi sarcoma	Stage 4	Metastasis	>12 Months	No			No	Yes	Yes	No	Yes	No	Yes	Yes
K176	Yes	Colorectal	Stage 4	Metastasis	>12 Months	No			No	Yes	Yes	No	No	No	No	Yes
K177	Yes	Pancreas	Stage 4	Metastasis	>12 Months	No			Yes	Yes	No	No	Yes	Yes	Yes	Yes
K178	Yes	Kaposi sarcoma	Stage 3	Lymph nodes	>12 Months	Yes	More than 6 times	Exclusive	No	Yes	Yes	No	Yes	Yes	No	Yes
K179	Yes	Osteosarcoma	Stage 3	Lymph nodes	6 Months	Yes	Less than 4 times	Exclusive	No	No	Yes	No	No	No	Yes	Yes
K180	Yes	Breast	Stage 3	Lymph nodes	>12 Months	Yes	More than 6 times	neo-Adjuvant	No	Yes	Yes	No	No	No	No	Yes
K181	Yes	Leukemia	Unknwon stage		6 Months	Yes	Less than 4 times	Exclusive	No	No	Yes	No	No	No	Yes	Yes
K182	Yes	Lung	Unknwon stage		6 Months	Yes	Less than 4 times	Exclusive	No	Yes	Yes	No	No	No	Yes	No
K183	Yes	Leukemia	Stage 2	Tumeur	6 Months	No			Yes	Yes	Yes	No	No	Yes	Yes	Yes
K184	Yes	Prostate	Stage 4	Metastasis	>12 Months	Yes	Less than 4 times	Exclusive	No	Yes	Yes	No	No	No	No	No
K185	Yes	Breast	Unknwon stage		12 Months	Yes	Less than 4 times	Exclusive	No	No	Yes	No	No	No	No	No
K186	Yes	Breast	Stage 3	Lymph nodes	12 Months	Yes	Less than 4 times	neo-Adjuvant	No	Yes	Yes	Yes	Yes	Yes	Yes	Yes
K187	Yes	Breast	Stage 4	Metastasis	>12 Months	Yes	More than 6 times	Palliative	Yes	Yes	Yes	Yes	Yes	Yes	Yes	Yes
K188	Yes	Pancreas	Stage 4	Metastasis	>12 Months	No			No	Yes	Yes	Yes	Yes	Yes	Yes	Yes
K189	Yes	Breast	Stage 4	Metastasis	>12 Months	No			No	Yes	Yes	No	No	Yes	No	Yes
K190	Yes	Breast	Stage 4	Metastasis	>12 Months	No			No	Yes	Yes	No	No	No	Yes	Yes
K191	Yes	Osteosarcoma	Unknwon stage		6 Months	Yes	Less than 4 times	Exclusive	No	No	Yes	No	No	No	No	Yes
K192	Yes	Cavum	Unknwon stage		>12 Months	Yes	More than 6 times	Exclusive	No	Yes	Yes	No	No	Yes	Yes	Yes
K193	Yes	Colorectal	Stage 4	Metastasis	>12 Months	No			No	Yes	Yes	No	No	No	No	No
K194	Yes	Breast	Stage 4	Metastasis	>12 Months	Yes	More than 6 times	Adjuvant	No	Yes	Yes	No	No	No	No	Yes
K195	Yes	Kaposi sarcoma	Stage 4	Metastasis	>12 Months	Yes	More than 6 times	Exclusive	No	No	Yes	No	No	No	No	No
K196	Yes	Osteosarcoma	Stage 4	Metastasis	>12 Months	Yes	More than 6 times	Exclusive	No	Yes	Yes	No	Yes	Yes	Yes	Yes
K197	Yes	Osteosarcoma	Stage 4	Metastasis	>12 Months	Yes	More than 6 times	Exclusive	No	Yes	Yes	No	No	No	No	No

K198	Yes	Breast	Stage 4	Metastasis	>12 Months	Yes	More than 6 times	neo-Adjuvant	No	Yes	Yes	No	No	No	No	Yes
K199	Yes	Lung	Stage 4	Metastasis	>12 Months	No			No	Yes	Yes	No	Yes	Yes	Yes	Yes
K200	Yes	Breast	Stage 4	Metastasis	>12 Months	Yes	Less than 4 times	neo-Adjuvant	No	No	Yes	No	No	No	No	Yes
K201	Yes	Colorectal	Stage 4	Metastasis	>12 Months	Yes	More than 6 times	neo-Adjuvant	No	No	Yes	No	No	No	No	No
K202	Yes	Osteosarcoma	Stage 4	Metastasis	>12 Months	No			No	Yes	Yes	No	Yes	Yes	Yes	Yes
K203	Yes	Osteosarcoma	Stage 3	Lymph nodes	>12 Months	No			Yes	Yes	No	No	No	Yes	Yes	Yes
K204	Yes	Cervical	Stage 4	Metastasis	>12 Months	Yes	Less than 4 times	Exclusive	No	No	Yes	No	No	No	No	No
K205	Yes	Cervical	Stage 4	Metastasis	>12 Months	No			Yes	Yes	No	No	No	Yes	No	Yes
K206	Yes	Breast	Stage 3	Lymph nodes	12 Months	Yes	More than 6 times	neo-Adjuvant	No	No	Yes	No	No	No	No	No
K207	Yes	Breast	Stage 3	Lymph nodes	12 Months	Yes	More than 6 times	neo-Adjuvant	No	No	Yes	No	No	No	Yes	Yes
K208	Yes	Breast	Stage 4	Metastasis	>12 Months	Yes	More than 6 times	neo-Adjuvant	No	Yes	Yes	Yes	No	No	No	Yes
K209	Yes	Colorectal	Stage 4	Metastasis	12 Months	Yes	Less than 4 times	Exclusive	No	Yes	Yes	No	No	No	No	No
K210	Yes	Cervical	Stage 3	Lymph nodes	12 Months	No			No	Yes	Yes	Yes	Yes	Yes	Yes	Yes
K211	Yes	Breast	Stage 3	Lymph nodes	6 Months	Yes	Less than 4 times	Exclusive	No	No	Yes	Yes	No	No	Yes	Yes
K212	Yes	Cavum	Stage 3	Lymph nodes	12 Months	Yes	Less than 4 times	Exclusive	Yes	No	No	Yes	No	No	Yes	Yes
K213	Yes	Breast	Stage 4	Metastasis	>12 Months	Yes	More than 6 times	neo-Adjuvant	No	Yes	Yes	No	No	No	No	No
K214	Yes	Breast	Unknwon stage		6 Months	Yes	Less than 4 times	neo-Adjuvant	No	Yes	Yes	No	No	No	Yes	No
K215	Yes	Breast	Stage 4	Metastasis	>12 Months	Yes	More than 6 times	Exclusive	No	Yes	Yes	No	Yes	Yes	Yes	No
K216	Yes	Breast	Unknwon stage		6 Months	Yes	Less than 4 times	neo-Adjuvant	No	Yes	Yes	No	Yes	Yes	Yes	Yes
K217	Yes	Cholangiocarcinoma	Stage 4	Metastasis	>12 Months	Yes	Less than 4 times	Exclusive	Yes	Yes	Yes	No	No	No	No	Yes
K218	Yes	Breast	Stage 4	Metastasis	12 Months	Yes	Less than 4 times	neo-Adjuvant	No	No	Yes	No	No	No	No	No
K219	Yes	Breast	Stage 3	Lymph nodes	6 Months	Yes	Less than 4 times	neo-Adjuvant	No	Yes	Yes	No	Yes	Yes	Yes	Yes
K220	Yes	Breast	Stage 4	Metastasis	6 Months	No			No	Yes	Yes	Yes	Yes	Yes	Yes	Yes
K221	Yes	Breast	Stage 4	Metastasis	12 Months	No			No	Yes	Yes	No	Yes	Yes	No	Yes
K222	Yes	Stomach	Stage 4	Metastasis	6 Months	Yes	Less than 4 times	neo-Adjuvant	Yes	Yes	No	Yes	No	No	No	Yes

K223	Yes	Cervical	Stage 3	Lymph nodes	6 Months	No			No	Yes	Yes	Yes	No	Yes	No	Yes
K224	Yes	Leukemia	Stage 4	Metastasis	12 Months	No			No	Yes	Yes	Yes	Yes	Yes	Yes	Yes
K225	Yes	Stomach	Unknwon stage		6 Months	Yes	Less than 4 times	neo-Adjuvant	No	Yes	Yes	No	No	No	No	Yes
K226	Yes	Cervical	Stage 3	Lymph nodes	6 Months	Yes	Less than 4 times	neo-Adjuvant	No	Yes	Yes	Yes	No	Yes	Yes	Yes
K227	Yes	Cervical	Stage 4	Metastasis	>12 Months	No			Yes	No	No	No	No	No	No	Yes
K228	Yes	Breast	Stage 4	Metastasis	6 Months	No			Yes	No	Yes	No	Yes	No	No	No
K229	Yes	Eusophagus	Unknwon stage		6 Months	Yes	Less than 4 times	neo-Adjuvant	No	No	Yes	Yes	No	No	No	Yes
K230	Yes	Cervical	Stage 3	Lymph nodes	12 Months	Yes	Less than 4 times	Exclusive	No	Yes	Yes	Yes	No	No	Yes	Yes
K231	Yes	Cervical	Stage 4	Metastasis	>12 Months	Yes	Less than 4 times	Exclusive	No	Yes	Yes	No	No	Yes	No	Yes
K232	Yes	Cervical	Stage 4	Metastasis	>12 Months	Yes	More than 6 times	Exclusive	No	No	Yes	No	Yes	Yes	No	Yes
K233	Yes	Osteosarcoma	Stage 4	Metastasis	12 Months	No			No	Yes	Yes	Yes	Yes	Yes	Yes	Yes
K234	Yes	Breast	Stage 4	Metastasis	>12 Months	No			No	Yes	Yes	Yes	No	No	Yes	Yes
K235	Yes	Cervical	Stage 4	Metastasis	12 Months	No			No	Yes	Yes	No	No	Yes	Yes	Yes
K236	Yes	Prostate	Stage 4	Metastasis	>12 Months	No			No	Yes	Yes	Yes	Yes	Yes	Yes	Yes
K237	Yes	Stomach	Stage 4	Metastasis	12 Months	No			No	Yes	Yes	Yes	No	No	No	Yes
K238	Yes	Eusophagus	Stage 3	Lymph nodes	6 Months	No			No	Yes	Yes	No	Yes	Yes	Yes	Yes
K239	Yes	Cervical	Stage 4	Metastasis	6 Months	No			No	Yes	Yes	No	No	Yes	Yes	Yes
K240	Yes	Stomach	Stage 4	Metastasis	12 Months	No			No	Yes	Yes	Yes	Yes	Yes	Yes	Yes
K241	Yes	Breast	Stage 4	Metastasis	12 Months	Yes	More than 6 times	Exclusive	Yes	No	Yes	No	Yes	Yes	Yes	Yes
K242	Yes	Breast	Stage 3	Lymph nodes	6 Months	Yes	Less than 4 times	neo-Adjuvant	No	Yes	Yes	No	No	No	Yes	Yes
K243	Yes	Ovary	Stage 4	Metastasis	12 Months	Yes	More than 6 times	Exclusive	No	Yes	Yes	Yes	Yes	Yes	Yes	Yes
K244	Yes	Breast	Stage 3	Lymph nodes	12 Months	Yes	More than 6 times	neo-Adjuvant	No	Yes	Yes	No	No	No	No	No
K245	Yes	Cervical	Stage 4	Metastasis	6 Months	Yes	Less than 4 times	neo-Adjuvant	No	Yes	Yes	Yes	Yes	Yes	Yes	Yes
K246	Yes	Ovary	Stage 4	Metastasis	>12 Months	Yes	More than 6 times	Palliative	No	Yes	Yes	No	Yes	No	Yes	Yes
K247	Yes	Breast	Stage 4	Metastasis	6 Months	No			No	Yes	Yes	No	No	No	No	Yes
K248	Yes	Liver	Stage 4	Metastasis	6 Months	No			No	Yes	Yes	No	Yes	Yes	Yes	Yes

K249	Yes	Pancreas	Unknwon stage		6 Months	Yes	More than 6 times	Adjuvant	No	No	Yes	No	No	No	No	No
K250	Yes	Cervical	Stage 4	Metastasis	6 Months	No			No	Yes	Yes	Yes	Yes	Yes	Yes	Yes
K251	Yes	Ovary	Stage 4	Metastasis	>12 Months	Yes	More than 6 times	Palliative	No	Yes	Yes	No	No	No	No	Yes
K252	Yes	Osteosarcoma	Stage 4	Metastasis	12 Months	Yes	Less than 4 times	neo-Adjuvant	No	Yes	Yes	No	No	No	No	Yes
K253	Yes	Eusophagus	Stage 4	Metastasis	6 Months	No			No	Yes	Yes	No	No	No	No	No
K254	Yes	Stomach	Stage 4	Metastasis	6 Months	Yes	Less than 4 times	neo-Adjuvant	Yes	No	Yes	No	No	No	Yes	Yes
K255	Yes	Prostate	Stage 4	Metastasis	>12 Months	Yes	Less than 4 times	Palliative	No	Yes	Yes	No	No	Yes	Yes	Yes
K256	Yes	Liver	Stage 4	Metastasis	6 Months	No			No	Yes	Yes	Yes	Yes	Yes	Yes	Yes
K257	Yes	Liver	Stage 4	Metastasis	12 Months	No			No	Yes	Yes	Yes	Yes	Yes	Yes	Yes
K258	Yes	Cervical	Stage 4	Metastasis	12 Months	No			No	Yes	Yes	Yes	Yes	Yes	Yes	Yes
K259	Yes	Ovary	Stage 4	Metastasis	6 Months	Yes	Less than 4 times	neo-Adjuvant	No	Yes	Yes	Yes	Yes	Yes	Yes	Yes
K260	Yes	Breast	Stage 3	Lymph nodes	12 Months	Yes	More than 6 times	Adjuvant	No	Yes	Yes	No	No	Yes	Yes	Yes
K261	Yes	Cervical	Unknwon stage		6 Months	Yes	Less than 4 times	neo-Adjuvant	No	Yes	Yes	No	No	No	No	Yes
K262	Yes	Eusophagus	Stage 3	Lymph nodes	6 Months	Yes	Less than 4 times	neo-Adjuvant	No	Yes	Yes	Yes	Yes	Yes	Yes	Yes
K263	Yes	Cervical	Stage 3	Lymph nodes	6 Months	Yes	Less than 4 times	neo-Adjuvant	No	Yes	Yes	No	No	No	Yes	Yes
K264	Yes	Eusophagus	Unknwon stage		6 Months	No			No	Yes	Yes	No	No	No	No	Yes
K265	Yes	Breast	Stage 3	Lymph nodes	6 Months	Yes	More than 6 times	neo-Adjuvant	No	Yes	Yes	Yes	Yes	Yes	Yes	Yes
K266	Yes	Breast	Stage 4	Metastasis	6 Months	Yes	More than 6 times	Palliative	No	Yes	Yes	Yes	Yes	No	Yes	Yes
K267	Yes	Kaposi sarcoma	Unknwon stage		>12 Months	Yes	Less than 4 times	Exclusive	No	Yes	Yes	Yes	Yes	Yes	Yes	Yes
K268	Yes	Breast	Stage 4	Metastasis	>12 Months	Yes	More than 6 times	Adjuvant	No	Yes	Yes	Yes	Yes	Yes	No	Yes
K269	Yes	Breast	Stage 4	Metastasis	>12 Months	Yes	More than 6 times	Adjuvant	No	Yes	Yes	No	No	Yes	Yes	Yes
K270	Yes	Pancreas	Stage 4	Metastasis	12 Months	Yes	More than 6 times	Adjuvant	Yes	Yes	Yes	Yes	Yes	Yes	Yes	Yes
K271	Yes	Breast	Stage 3	Lymph nodes	12 Months	Yes	More than 6 times	neo-Adjuvant	No	Yes	Yes	Yes	No	No	Yes	Yes
K272	Yes	Cervical	Stage 4	Metastasis	12 Months	No			No	Yes	Yes	No	No	Yes	Yes	Yes
K273	Yes	Breast	Stage 4	Metastasis	6 Months	No			No	Yes	Yes	No	Yes	Yes	Yes	Yes

K274	Yes	Cervical	Stage 4	Metastasis	12 Months	Yes	More than 6 times	Exclusive	No	Yes	Yes	Yes	Yes	Yes	Yes	Yes
K275	Yes	Breast	Stage 4	Metastasis	>12 Months	Yes	More than 6 times	Adjuvant	Yes	Yes	No	No	Yes	Yes	Yes	Yes
K276	Yes	Breast	Stage 4	Metastasis	6 Months	Yes	More than 6 times	neo-Adjuvant	No	No	Yes	No	No	No	No	No
K277	Yes	Breast	Stage 4	Metastasis	6 Months	Yes	More than 6 times	Adjuvant	No	Yes	Yes	No	No	Yes	Yes	Yes
K278	Yes	Lung	Stage 3	Lymph nodes	6 Months	Yes	More than 6 times	neo-Adjuvant	No	Yes	Yes	Yes	Yes	Yes	Yes	Yes
K279	Yes	Breast	Stage 4	Metastasis	6 Months	Yes	More than 6 times	Adjuvant	No	Yes	Yes	Yes	Yes	Yes	Yes	Yes
K280	Yes	Liver	Stage 4	Metastasis	6 Months	No			No	Yes	Yes	Yes	Yes	Yes	Yes	Yes
K281	Yes	Breast	Stage 3	Lymph nodes	>12 Months	Yes	More than 6 times	Adjuvant	No	Yes	Yes	No	No	No	Yes	Yes
K282	Yes	Breast	Stage 4	Metastasis	6 Months	No			No	Yes	Yes	No	No	Yes	No	Yes
K283	Yes	Kaposi sarcoma	Stage 4	Metastasis	6 Months	No			No	Yes	Yes	No	No	No	No	Yes
K284	Yes	Cervical	Stage 4	Metastasis	6 Months	No			No	Yes	Yes	No	No	No	Yes	Yes
K285	Yes	Cervical	Stage 4	Metastasis	6 Months	No			No	Yes	Yes	No	No	No	No	No
K286	Yes	Liver	Stage 4	Metastasis	>12 Months	No			No	Yes	Yes	No	Yes	Yes	Yes	Yes
K287	Yes	Stomach	Stage 4	Metastasis	6 Months	Yes	More than 6 times	Palliative	No	Yes	Yes	Yes	Yes	Yes	Yes	Yes
K288	Yes	Breast	Unknwon stage		6 Months	Yes	More than 6 times	neo-Adjuvant	No	Yes	Yes	No	No	No	No	No
K289	Yes	Lung	Stage 3	Lymph nodes	6 Months	Yes	Less than 4 times	Exclusive	Yes	Yes	No	No	No	No	No	Yes
K290	Yes	Pancreas	Stage 4	Metastasis	12 Months	Yes	More than 6 times	Exclusive	No	No	Yes	No	No	No	No	Yes
K291	Yes	Colorectal	Stage 3	Lymph nodes	6 Months	Yes	More than 6 times	Adjuvant	No	Yes	Yes	Yes	Yes	Yes	Yes	Yes
K292	Yes	Kaposi sarcoma	Stage 4	Metastasis	>12 Months	Yes	More than 6 times	Exclusive	No	Yes	Yes	No	Yes	No	Yes	Yes
K293	Yes	Breast	Stage 4	Metastasis	6 Months	No			No	Yes	Yes	No	Yes	Yes	Yes	Yes
K294	Yes	Cervical	Stage 4	Metastasis	6 Months	Yes	More than 6 times	Exclusive	No	Yes	Yes	Yes	No	No	No	Yes
K295	Yes	Kaposi sarcoma	Stage 3	Lymph nodes	6 Months	Yes	Less than 4 times	Exclusive	No	Yes	Yes	No	No	No	No	No
K296	Yes	Breast	Stage 3	Lymph nodes	12 Months	Yes	More than 6 times	neo-Adjuvant	Yes	No	No	No	Yes	Yes	Yes	Yes
K297	Yes	Pancreas	Stage 4	Metastasis	>12 Months	Yes	More than 6 times	Adjuvant	No	Yes	Yes	No	No	No	No	Yes
K298	Yes	Breast	Unknwon stage		6 Months	Yes	Less than 4 times	neo-Adjuvant	No	Yes	Yes	No	No	No	No	No

K299	Yes	Pancreas	Stage 4	Metastasis	12 Months	No			No	Yes	Yes	Yes	Yes	Yes	Yes	Yes
K300	Yes	Prostate	Stage 4	Metastasis	12 Months	No			No	Yes	Yes	No	Yes	Yes	Yes	Yes
K301	Yes	Osteosarcoma	Stage 4	Metastasis	6 Months	No			Yes	Yes	No	No	No	No	Yes	Yes
K302	Yes	Liver	Stage 4	Metastasis	12 Months	No			No	Yes	Yes	No	No	No	No	No
K303	Yes	Ovary	Stage 4	Metastasis	12 Months	No			No	Yes	No	No	No	No	No	Yes
K304	Yes	Ovary	Stage 4	Metastasis	6 Months	Yes	Less than 4 times	Palliative	No	Yes	Yes	No	No	No	No	No
K305	Yes	Breast	Stage 4	Metastasis	12 Months	Yes	More than 6 times	Adjuvant	Yes	Yes	No	Yes	No	Yes	Yes	Yes
K306	Yes	Leukemia	Stage 4	Metastasis	6 Months	Yes	More than 6 times	Exclusive	No	No	Yes	Yes	Yes	Yes	Yes	Yes
K307	Yes	Breast	Stage 4	Metastasis	>12 Months	Yes	More than 6 times	Exclusive	Yes	Yes	Yes	No	Yes	Yes	No	Yes
Legend: M, male; F, female; code with K, cancer patient;																

S3. Patients, *S. aureus infection, and antibiogram*

**Patients**	**Bacteria, antibiotics, and susceptibility profile**																	
**Patient Code**	**Staphylococcal infection?**	**IMP**	**AMX**	**CAZ**	**FOX**	**AMC**	**CXM**	**COT**	**ERY**	**AMK**	**GEN**	**CIP**	**OFX**	**FUS**	**TET**	**OXA**	**VAN**	**NIT**	**MRSA**
K001	Yes	S	R	R	R	R	R	R	R	R	S	R	R	S	R	R	R	R	Yes
K002	No																		
K003	No																		
K004	No																		
K005	No																		
K006	No																		
K007	Yes	R	R	R	R	R	R	S	R	R	S	R	R	S	R	R	R	R	Yes
K008	No																		
K009	No																		
K010	No																		
K011	No																		
K012	No																		
K013	No																		
K014	Yes	S	R	R	R	R	R	R	R	S	S	R	R	R	R	R	R	R	Yes
K015	No																		
K016	No																		
K017	No																		
K018	No																		
K019	Yes	S	R	R	R	R	R	R	R	S	S	R	R	R	R	R	R	R	Yes
K020	Yes	S	S	R	R	R	R	S	R	S	S	R	R	R	R	R	R	R	Yes
K021	No																		
K022	No																		
K023	No																		
K024	No																		
K025	No																		

K026	Yes	S	R	R	S	S	R	S	S	R	S	R	R	R	R	R	R	R	No
K027	Yes	S	R	R	R	R	R	R	R	R	R	R	R	S	R	R	R	R	Yes
K028	No																		
K029	No																		
K030	No																		
K031	No																		
K032	No																		
K033	No																		
K034	No																		
K035	No																		
K036	No																		
K037	No																		
K038	No																		
K039	No																		
K040	No																		
K041	Yes	S	R	R	R	R	R	R	S	S	S	R	R	R	R	R	R	R	Yes
K042	Yes	S	R	R	R	R	R	R	R	S	R	R	R	S	R	R	S	R	Yes
K043	No																		
K044	No																		
K045	No																		
K046	No																		
K047	No																		
K048	No																		
K049	No																		
K050	Yes	R	R	R	R	R	R	R	R	S	S	R	R	R	S	R	S	R	Yes
K051	No																		
K052	No																		
K053	Yes	S	S	R	R	R	R	S	R	S	R	S	S	S	R	R	R	R	Yes
K054	No																		

K055	No																		
K056	No																		
K057	No																		
K058	No																		
K059	No																		
K060	Yes	S	R	R	R	R	R	R	S	R	S	R	R	R	R	R	R	R	Yes
K061	No																		
K062	No																		
K063	No																		
K064	No																		
K065	Yes	S	S	R	S	R	S	R	S	S	S	S	S	R	S	R	R	R	No
K066	No																		
K067	No																		
K068	No																		
K069	No																		
K070	No																		
K071	No																		
K072	No																		
K073	No																		
K074	No																		
K075	Yes	S	R	R	R	R	R	S	R	R	S	R	R	R	R	R	R	R	Yes
K076	No																		
K077	No																		
K078	No																		
K079	No																		
K080	No																		
K081	No																		
K082	No																		
K083	No																		

K084	No																		
K085	No																		
K086	Yes	S	S	S	S	S	S	S	R	S	S	S	S	R	R	R	R	R	No
K087	No																		
K088	No																		
K089	No																		
K090	Yes	S	S	S	R	R	R	R	R	S	R	R	R	R	R	R	R	R	Yes
K091	Yes	S	R	R	R	R	R	R	R	S	S	R	R	R	R	R	R	R	Yes
K092	No																		
K093	Yes	S	S	R	R	R	R	R	R	S	S	R	R	R	R	R	R	R	Yes
K094	No																		
K095	Yes	S	S	R	R	R	R	R	R	S	S	R	R	R	R	R	R	R	Yes
K096	No																		
K097	No																		
K098	No																		
K099	No																		
K100	Yes	S	R	S	S	S	R	S	R	S	S	R	R	S	R	R	R	R	No
K101	No																		
K102	No																		
K103	Yes	S	S	R	S	S	R	S	R	S	S	S	S	R	R	R	R	R	No
K104	No																		
K105	No																		
K106	Yes	S	R	R	R	R	R	R	R	S	S	R	R	R	S	R	R	S	Yes
K107	Yes	S	R	R	R	R	R	R	R	S	S	R	R	R	R	R	R	R	Yes
K108	Yes	S	R	S	R	R	S	S	R	S	S	R	S	S	R	R	R	R	Yes
K109	Yes	S	R	S	R	S	R	R	R	S	S	R	R	R	R	R	R	R	Yes
K110	Yes	S	R	R	R	R	R	R	R	R	R	R	R	R	R	R	R	R	Yes
K111	Yes	S	R	R	R	R	R	S	R	S	S	R	S	R	R	R	R	R	Yes
K112	No																		

K113	No																		
K114	No																		
K115	No																		
K116	No																		
K117	Yes	S	R	R	S	S	R	R	R	S	S	R	S	R	R	R	R	R	No
K118	Yes	S	S	R	R	R	R	S	R	S	S	R	S	S	S	R	S	S	Yes
K119	No																		
K120	Yes	S	R	R	R	R	R	S	R	S	S	R	R	R	R	R	R	R	Yes
K121	No																		
K122	No																		
K123	No																		
K124	No																		
K125	Yes	S	R	R	R	R	R	R	R	S	R	R	R	R	R	R	R	R	Yes
K126	No																		
K127	No																		
K128	Yes	S	R	R	R	R	R	S	R	S	S	S	S	R	R	R	R	R	Yes
K129	No																		
K130	No																		
K131	Yes	S	R	R	R	R	R	R	R	S	R	R	R	R	R	R	R	R	Yes
K132	Yes	S	R	R	R	R	R	R	R	S	S	R	R	R	R	R	R	R	Yes
K133	No																		
K134	No																		
K135	No																		
K136	No																		
K137	No																		
K138	Yes	S	S	R	R	R	R	S	R	S	S	R	R	S	R	R	R	R	Yes
K139	No																		
K140	No																		
K141	No																		

K142	No																		
K143	No																		
K144	No																		
K145	No																		
K146	No																		
K147	No																		
K148	No																		
K149	No																		
K150	No																		
K151	Yes	S	S	R	R	S	R	S	S	S	S	S	S	S	S	R	R	R	Yes
K152	No																		
K153	No																		
K154	No																		
K155	No																		
K156	No																		
K157	No																		
K158	No																		
K159	No																		
K160	No																		
K161	Yes	S	R	R	R	S	S	S	S	S	S	S	S	S	S	R	R	S	Yes
K162	No																		
K163	No																		
K164	No																		
K165	No																		
K166	No																		
K167	No																		
K168	No																		
K169	No																		
K170	No																		

K171	No																		
K172	No																		
K173	Yes	S	S	R	R	R	R	S	R	S	S	R	R	R	S	R	S	R	Yes
K174	Yes	S	R	S	R	R	S	R	R	R	R	S	R	R	R	R	R	R	Yes
K175	No																		
K176	No																		
K177	Yes	S	R	R	R	R	R	R	R	S	S	R	R	R	R	R	R	R	Yes
K178	No																		
K179	No																		
K180	No																		
K181	No																		
K182	No																		
K183	No																		
K184	No																		
K185	No																		
K186	No																		
K187	Yes	S	R	R	R	R	R	R	R	S	S	R	R	R	R	R	R	R	Yes
K188	No																		
K189	Yes	S	R	R	R	R	S	S	R	S	S	R	R	R	R	R	R	R	Yes
K190	Yes	S	S	S	S	S	R	S	R	S	S	S	S	I	R	R	R	R	No
K191	No	S	S	S	S	R	S	S	S	S	S	S	S	S	S	S	S	R	No
K192	Yes	S	S	R	S	R	R	S	R	S	S	R	R	R	R	R	R	R	No
K193	Yes	S	S	R	R	R	R	R	R	S	S	R	R	S	R	R	R	R	Yes
K194	No																		
K195	Yes	R	R	R	R	R	R	R	R	S	S	R	R	R	R	R	R	R	Yes
K196	No																		
K197	No																		
K198	No																		
K199	Yes	S	S	R	S	R	R	S	R	S	S	S	S	S	R	R	R	R	No

K200	Yes	R		S	S	R	S	S	R	S	S	S	S	S	R	R	R	R	No
K201	No																		
K202	No																		
K203	Yes	S	R	R	R	S	R	R	R	S	S	R	S	R	R	R	R	R	Yes
K204	No																		
K205	No																		
K206	No																		
K207	No																		
K208	No																		
K209	No																		
K210	No																		
K211	No																		
K212	No																		
K213	No																		
K214	No																		
K215	No																		
K216	No																		
K217	No																		
K218	No																		
K219	No																		
K220	No																		
K221	No																		
K222	No																		
K223	No																		
K224	No																		
K225	No																		
K226	No																		
K227	No																		
K228	No																		

K229	No																		
K230	No																		
K231	No																		
K232	No																		
K233	No																		
K234	No																		
K235	No																		
K236	No																		
K237	No																		
K238	No																		
K239	No																		
K240	No																		
K241	No																		
K242	No																		
K243	No																		
K244	No																		
K245	No																		
K246	No																		
K247	No																		
K248	No																		
K249	Yes	S	R	R	R	R	R	R	R	S	S	I	I	R	R	R	R	R	Yes
K250	No																		
K251	No																		
K252	No																		
K253	No																		
K254	No																		
K255	No																		
K256	No																		
K257	No																		

K258	No																		
K259	No																		
K260	Yes	S	S	R	S	S	S	S	S	S	S	R	R	R	S	S	S	S	No
K261	No	S	R	R	R	R	R	R	R	S	S	R	R	R	R	R	R	R	Yes
K262	Yes	S	R	R	R	S	R	R	R	R	S	R	R	R	R	R	R	R	Yes
K263	No																		
K264	No																		
K265	No																		
K266	No																		
K267	No																		
K268	No																		
K269	No																		
K270	No																		
K271	No																		
K272	Yes	S	R	R	R	R	R	R	R	S	S	I	I	S	R	R	R	R	Yes
K273	No																		
K274	No																		
K275	No																		
K276	No																		
K277	No																		
K278	No																		
K279	No																		
K280	No																		
K281	No																		
K282	No																		
K283	No																		
K284	Yes	S	R	R	R	S	S	S	R	S	S	R	R	R	R	R	R	R	Yes
K285	No																		
K286	No																		

K287	No																		
K288	Yes	R	R	R	R	R	R	R	R	R	S	R	R	R	R	R	R	R	Yes
K289	Yes	S	R	R	R	R	S	S	R	S	S	R	R	R	R	R	R	R	Yes
K290	No																		
K291	No																		
K292	Yes	S	R	R	R	S	R	S	S	S	S	S	S	S	R	R	R	R	Yes
K293	No																		
K294	No																		
K295	No																		
K296	No																		
K297	Yes	S	R	R	R	R	R	R	R	R	S	R	R	R	R	R	R	R	Yes
K298	No																		
K299	Yes	S	R	S	R	R	R	S	R	S	S	R	R	R	R	R	R	R	Yes
K300	Yes	S	R	R	R	R	R	R	R	R	R	R	R	R	R	R	R	R	Yes
K301	No																		
K302	No																		
K303	No																		
K304	No																		
K305	No																		
K306	No																		
K307	No																		
**Non-cancer patient**																			
D001	No																		
D002	No																		
D003	No																		
D004	No																		
D005	No																		

D006	No																		
D007	No																		
D008	No																		
D009	Yes	S	R	S	S	S	S	S	R	S	S	S	S	S	S	S	R	I	No
D010	No																		
D011	No																		
D012	No																		
D013	No																		
D014	No																		
D015	No																		
D016	No																		
D017	No																		
D018	Yes	S	R	R	R	R	R	R	R	S	S	S	S	R	R	R	R	R	Yes
D019	No																		
D020	Yes	S	R	R	R	S	R		R	R	S	R	R	S	S	S	R	S	Yes
D021	No																		
D022	No																		
D023	No																		
D024	No																		
D025	Yes	S	R	R	R	S	R	S	R	S	S	S	S	R	R	R	R	R	Yes
D026	No																		
D027	No																		
D028	No																		
D029	Yes	S	S	S	S	R	S	S	S	S	S	S	S	S		S	S	S	No
D030	No																		
D031	Yes	R	S	S	R	R	R	R	R	S	S	S	S	S	R	R	R	S	Yes
D032	No																		
D033	No																		
D034	No																		

D035	No																		
D036	No																		
D037	No																		
D038	No																		
D039	Yes	S	R	R	R	R	R	R	R	S	S	S	S		R	R	R	R	Yes
D040	No																		
D041	Yes	S	R	R	R	R	R	R	R	R	S	S	S	S	R	R	R	R	Yes
D042	No																		
D043	No																		
D044	No																		
D045	No																		
D046	No																		
D047	Yes	S	R	R	S	S	R	R	R	S	S	S	S	S	R	R	R	S	No
D048	Yes	S	R	R	R	S	R	R		S	S	R	R	R	R	R	R	S	Yes
D049	No								R	S	S	I	I	R	R			S	
D050	Yes	S	R	R	S	S	S	S	R	S	S	R	S	R	R	R	R	S	No
D051	Yes	S	R	S	R	S	R	S	R	S	S	R	R	R	R	R	R	R	Yes
D052	Yes	S	R	R	S	S		R	R	S	S	I	R	R	R			S	No
D053	No																		
D054	No			R	R	R		R	R	R	R	S	S	R	R	R	R	S	Yes
D055	Yes	R	R	R	R	S	R	R	R	S	S	R	R	R	R	R	R	R	Yes
D056	No																		
D057	Yes	R	R	S	R	S	R	S	R	S	S	I	R	S	S	S	R	S	Yes
D058	Yes	S	R	R	S	S	R	S				S	S	S	R	R	R	S	No
D059	No																		
D060	Yes	S	R	R	S	S	S	R	R	S	S	S	S	R	R	R	R	R	No
D061	No																		
D062	No																		
D063	No																		

D064	No																		
D065	No																		
D066	No																		
D067	No																		
D068	No																		
D069	No																		
D070	No																		
D071	No																		
D072	No																		
D073	No																		
D074	Yes	S	R	R	R	S	R	R	R	R	R	S	S	R	R	S	S	S	Yes
D075	No																		
D076	No																		
D077	No	Legends: MRSA, methicillin-resistant Staphylococcus aureus; AMX: Amoxicillin, AMC: Amoxicillin -clavulanic acid, CAZ: Ceftazidime, FOX: Cefoxitin, AMK: Amikacin, GEN: Gentamicin, TET: Tetracycline, CIP: Ciprofloxacin, NIT: Nitrofurantoin, OFX: Ofloxacin, COT: Trimethoprim-sulfamethoxazole, FUS: Fusidic acid, ERY: Erythromycin.																	
D078	No																		
D079	No																		
D080	No																		
D081	No																		
D082	No																		
D083	No	R: Resistant; S: Sensitive; I: Intermediate																	
D084	No																		
D085	No																		
D086	No																		
D087	No																		
D088	No																		
D089	No																		
D090	No																		
D091	No																		

D092	No																		
D093	No																		
D094	No																		
D095	No																		
D096	No																		
D097	No																		
D098	No																		
D099	No																		
D100	No																		
D101	No																		
D102	No																		
D103	No																		
D104	No																		
D105	No																		
D106	No																		
D107	No																		
D108	No																		
D109	No																		
D110	No																		
D111	No																		
D112	No																		
D113	No																		
D114	No																		
D115	No																		
D116	No																		
D117	No																		
D118	No																		
D119	No																		
D120	No																		

D121	No																		
D122	No																		
D123	No																		
D124	No																		
D125	No																		
D126	No																		
D127	No																		
D128	No																		
D129	No																		
D130	No																		
D131	No																		
D132	No																		
D133	No																		
D134	No																		
D135	No																		
D136	No																		
D137	No																		
D138	No																		
D139	No																		
D140	No																		
D141	No																		
D142	No																		
D143	No																		
D144	No																		
D145	No																		
D146	No																		
D147	No																		
D148	No																		
D149	No																		

D150	No																		
D151	No																		
D152	No																		
D153	No																		
D154	No																		
D155	No																		
D156	No																		
D157	No																		
D158	No																		
D159	No																		
D160	No																		
D161	No																		
D162	No																		
D163	No																		
D164	No																		
D165	No																		
D166	No																		
D167	No																		
D168	No																		
D169	No																		
D170	No																		
D171	No																		
D172	No																		
D173	No																		
D174	No																		
D175	No																		
D176	No																		
D177	No																		
D178	No																		

D179	No																		
D180	No																		
D181	No																		
D182	No																		
D183	No																		
D184	No																		
D185	No																		
D186	No																		
D187	No																		
D188	No																		
D189	No																		
D190	No																		
D191	No																		
D192	No																		
D193	No																		
D194	No																		
D195	No																		
D196	No																		
D197	No																		
D198	No																		
D199	No																		
D200	No																		

Legend: M, male; F, female; code with K, cancer patient; code with D, non-cancer patient
